# Difference in respiratory syncytial virus-specific Fc-mediated antibody effector functions between children and adults

**DOI:** 10.1093/cei/uxad101

**Published:** 2023-08-22

**Authors:** Anke J Lakerveld, Anne T Gelderloos, Rutger M Schepp, Cornelis A M de Haan, Robert S van Binnendijk, Nynke Y Rots, Josine van Beek, Cécile A C M van Els, Puck B van Kasteren

**Affiliations:** Center for Immunology of Infectious Diseases and Vaccines, Center for Infectious Disease Control, National Institute for Public Health and the Environment (RIVM), Bilthoven, The Netherlands; Department of Medical Microbiology, Leiden University Medical Center, The Netherlands; Center for Immunology of Infectious Diseases and Vaccines, Center for Infectious Disease Control, National Institute for Public Health and the Environment (RIVM), Bilthoven, The Netherlands; Center for Immunology of Infectious Diseases and Vaccines, Center for Infectious Disease Control, National Institute for Public Health and the Environment (RIVM), Bilthoven, The Netherlands; Section Virology, Department Biomolecular Health Sciences, Faculty Veterinary Medicine, Utrecht University, The Netherlands; Center for Immunology of Infectious Diseases and Vaccines, Center for Infectious Disease Control, National Institute for Public Health and the Environment (RIVM), Bilthoven, The Netherlands; Center for Immunology of Infectious Diseases and Vaccines, Center for Infectious Disease Control, National Institute for Public Health and the Environment (RIVM), Bilthoven, The Netherlands; Center for Immunology of Infectious Diseases and Vaccines, Center for Infectious Disease Control, National Institute for Public Health and the Environment (RIVM), Bilthoven, The Netherlands; Center for Immunology of Infectious Diseases and Vaccines, Center for Infectious Disease Control, National Institute for Public Health and the Environment (RIVM), Bilthoven, The Netherlands; Section Immunology, Department Biomolecular Health Sciences, Faculty of Veterinary Medicine, Utrecht University, The Netherlands; Center for Immunology of Infectious Diseases and Vaccines, Center for Infectious Disease Control, National Institute for Public Health and the Environment (RIVM), Bilthoven, The Netherlands

**Keywords:** antibodies, anti-viral immunity, phagocytosis, complement, cytotoxicity

## Abstract

Respiratory syncytial virus (RSV) infections are a major cause of bronchiolitis and pneumonia in infants and older adults, for which there is no known correlate of protection. Increasing evidence suggests that Fc-mediated antibody effector functions have an important role, but little is known about the development, heterogeneity, and durability of these functional responses. In light of future vaccine strategies, a clear view of the immunological background and differences between various target populations is of crucial importance.

In this study, we have assessed both quantitative and qualitative aspects of RSV-specific serum antibodies, including IgG/IgA levels, IgG subclasses, antibody-dependent complement deposition, cellular phagocytosis, and NK cell activation (ADNKA). Samples were collected cross-sectionally in different age groups (11-, 24-, and 46-month-old children, adults, and older adults; *n* = 31–35 per group) and longitudinally following natural RSV infection in (older) adults (2–36 months post-infection; *n* = 10).

We found that serum of 24-month-old children induces significantly lower ADNKA than the serum of adults (*P* < 0.01), which is not explained by antibody levels. Furthermore, in (older) adults we observed boosting of antibody levels and functionality at 2–3 months after RSV infection, except for ADNKA. The strongest decrease was subsequently observed within the first 9 months, after which levels remained relatively stable up to three years post-infection.

Together, these data provide a comprehensive overview of the functional landscape of RSV-specific serum antibodies in the human population, highlighting that while antibodies reach adult levels already at a young age, ADNKA requires more time to fully develop.

## Teaser

Serum antibodies of children aged 24- and 46-months induce lower RSV-specific antibody-dependent NK cell activation compared to (older) adults. In contrast, no difference is found in RSV-specific IgG levels, antibody-dependent cellular phagocytosis, and antibody-dependent complement deposition.

## Graphical Abstract

**Graphical Abstract:**
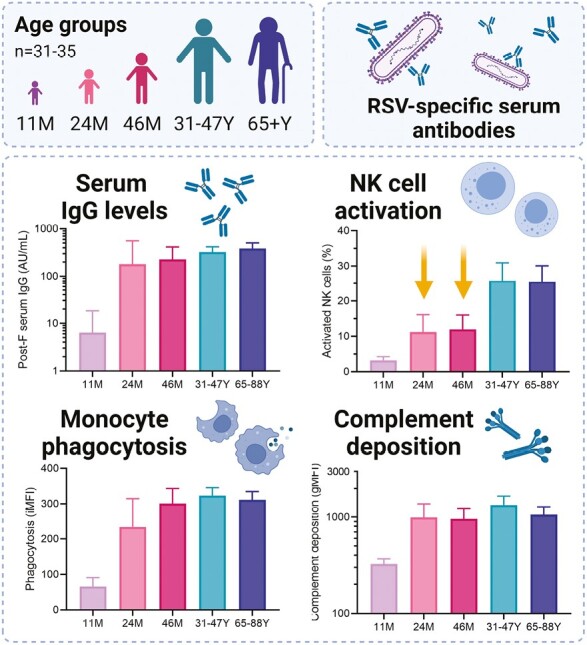

## Introduction

Respiratory syncytial virus (RSV) infections mostly result in mild or asymptomatic disease, but especially in infants and older adults, RSV may cause severe disease resulting in hospitalization or even death. Worldwide, an estimated 3.2 million children <5 years of age and an estimated 336 000 older adults (>65 years of age) were admitted to the hospital with an RSV-associated respiratory tract infection in 2015 [1, 2]. RSV re-infection in the absence of substantial antigenic change is common even in healthy adults, which is indicative of suboptimal immune protection upon natural infection [3, 4]. Currently, options for prevention are limited to the monoclonal antibody palivizumab or its recently approved successor nirsevimab, for use in (high-risk) infants only [5–7]. Importantly, various vaccines aiming to protect vulnerable groups via vaccination of infants/children, pregnant women, or older adults are in (late-stage) clinical development and several have recently been approved for marketing [8–10]. In light of the implementation and future improvement of these first-generation RSV vaccines, it is of pivotal importance to understand the immunological mechanisms underlying protection and disease, including the potential differences in correlates of protection between various target groups.

To date, correlates of protection for RSV disease remain poorly defined. In the Dutch population, it has been shown that mean RSV antibody levels remain stable from 5 up to 90 years of age [11]. Some studies show that high serum antibody titers protect—mainly in older adults—against RSV disease [12–14], while other studies do not show protection, even with relatively high antibody titers [15–19]. These studies all focus on either antibody binding or neutralization capacity. However, in addition to neutralization, antibodies can mediate other, Fc-dependent, effector functions which may either contribute to protection or play a role in pathogenesis. The infamous 1960s formalin-inactivated RSV vaccine induced poorly neutralizing antibodies, and it has been suggested that these were involved in enhanced disease upon natural infection [20, 21]. In contrast, it has recently been shown that in both non-human primates and humans, Fc-mediated antibody effector functions are important in protecting against RSV infection [22, 23].

The most well-known Fc-mediated antibody effector functions are antibody-mediated complement deposition (ADCD), antibody-dependent cellular phagocytosis (ADCP), and antibody-dependent cellular cytotoxicity (ADCC). An extensive overview of these effector functions in the context of RSV has been provided by van Erp *et al*. [24]. In short, the classical complement pathway can be activated through the recognition of antibody-antigen complexes, resulting in the deposition of among others complement factor C3b on pathogens and infected cells (ADCD). Activation of the complement cascade can lead to killing of the pathogen or infected cell by the complement system itself, or through phagocytosis by attracted immune cells. Antibodies can also be recognized directly by immune cells expressing Fc-receptors, which can subsequently phagocytose the opsonized pathogen or infected cell (ADCP) or release granules with cytotoxic contents that kill the infected cells (ADCC). Assays probing ADCC often use antibody-dependent natural killer cell activation (ADNKA) as a proxy for cytotoxicity.

To summarize, previous research has indicated that Fc-mediated antibody effector functions likely play an important role in immunological protection from RSV infection. However, limited information is available on the development, heterogeneity, and durability of these functionalities across the human population. In the current study, we have assessed both quantitative and qualitative aspects of RSV-specific serum antibodies (e.g. IgG/IgA binding titers, ADCD, ADCP, ADNKA) cross-sectionally in different age groups (11-, 24-, and 46-months, adults, and older adults; *n* = 31–35 per group) and longitudinally following natural RSV infection in (older) adults (2–36 months post-infection; *n* = 10). Together, these data provide a comprehensive overview of the functional landscape of RSV-specific serum antibodies in the human population that will support further research into much needed correlates of protection for this important pathogen.

## Methods

### Ethics statement

Participants were included from a total of four clinical studies performed in the Netherlands. For comparison between age groups, two studies with a primary aim of assessing pneumococcal carriage in children of 11, 24, and 46 months of age and parents (“OKIDOKI”) and one study of influenza vaccination in adults >60 years of age (“ILI”) were used (trialsearch.who.int: NTR3614, NTR5405, NTR3386). These studies were approved by the Medical Ethical Committee (METC) Noord Holland. For longitudinal evaluation of antibody functionality, the Immfact study was used (CCMO nr: NL46795.094.13). This study was approved by the METC Noord-Holland followed by management by the METC MEC-U (Utrecht, the Netherlands).

Peripheral blood mononuclear cells (PBMCs) for NK cell isolation for the ADNKA assay were obtained at the National Institute for Public Health and the Environment (RIVM, the Netherlands) from blood samples of healthy adult volunteers. Blood samples were processed anonymously and the research goal, primary cell isolation, required no review by an accredited Medical Research Ethics Committee (MREC), as determined by the Dutch Central Committee on Research involving human subjects (CCMO).

Written informed consent was obtained from all participants or their parents. This study was conducted according to good clinical practice, which includes the provisions of the Declaration of Helsinki.

### Study design

To compare antibody effector functions between age groups, serum samples were selected from 11- (*n* = 33), 24- (*n* = 31), and 46-month-old children (*n* = 35) and adults (31–47 years; *n* = 35) from the cross-sectional OKIDOKI studies [25]. Samples were collected during the winter of 2012/2013, except for the 46-month-old samples which were collected in the winter of 2015/2016 by repeated sampling of the 11-month-old group from 2012/2013. Of note, the selection used for this study contained paired samples for only 13 participants. Exclusion criteria were known or suspected immunodeficiency, craniofacial or chromosomal abnormalities, coagulation disorders, or use of anticoagulant medication. Adults with a BMI > 35 were also excluded. In addition, serum samples from the ILI study were selected (≥65 years of age; *n* = 35). This study comprised a prospective observational cohort study performed during the winter of 2012/2013 in older adults as previously described [26]. There were no exclusion criteria for this study. None of the selected participants had an ongoing RSV infection at the time of sampling as determined by PCR on nasal swab samples. Sample selection from all clinical cohorts for the purpose of this study was based on availability of material, maintaining equal sex ratios, equal distribution over the winter period and ages, and negative PCR for RSV during sampling. The OKIDOKI and ILI studies were conducted in parallel by design to allow for combined analysis. Participant characteristics can be found in Table 1.

**Table 1.** Participant characteristics.

Age groups (cross-sectional)	Female (%)	Mean age (range)
11 months (*n* = 33)	17 (51%)	328 days (311–350)
24 months (*n* = 31)	15 (48%)	735 days (704–757)
46 months (*n* = 35)	18 (51%)	1379 days (1339–1449)
Adults (*n* = 35)	18 (51%)	37 years (31–47)
Older adults (*n* = 35)	18 (51%)	75 years (65–88)

Post-infection kinetics (longitudinal)	Female (%)	Mean age (range)
RSV-confirmed adults and older adults (*n* = 10)	7 (70%)	61 years (45–87)
Age-matched controls (*n* = 10)	7 (70%)	60 years (46–79)

To investigate the kinetics of RSV antibody functionality post-infection, serum samples of the longitudinal Immfact study were used [27]. Participants were included in this study after symptomatic RSV infection for which a general practitioner was consulted and serum samples were collected 2-3, 9, 18, and 36 months post PCR-confirmed diagnosis from (older) adults (45–87 years; *n* = 10). Participants were diagnosed with RSV in the winter of 2016/2017 and longitudinal samples were collected from 2016 to 2020. In the same study, samples from age-matched healthy adult controls (46–79 years; *n* = 10) were collected in the years 2015, 2018, and 2019. Exclusion criteria for this study were being under sustained immunosuppressive therapy, having a known primary or secondary immunodeficiency, and having a bleeding disorder. Participant characteristics can be found in Table 1.

### Cells and viruses

THP-1 monocytic cells (ATCC TIB-202) were cultured in ATCC-modified Roswell Park Memorial Institute (RPMI)-1640 medium (30-2001; Gibco, Thermo Fisher Scientific, Waltham) containing 2 mM l-glutamine, 10 mM HEPES, 1 mM sodium pyruvate, 4500 mg/l glucose, and 1500 mg/l sodium bicarbonate, supplemented with 10% heat-inactivated fetal bovine serum (hiFBS; HyClone, Fisher Scientific), 1% penicillin/streptomycin/glutamine (PSG; cat#10378016, Gibco), and 0.05 mM 2-mercapto-ethanol. Cells were cultured at concentrations between 0.2 and 0.8 × 10^6^ cells/ml to maintain phagocytic activity of the cells. Vero cells (ATCC CCL-81) were cultured in Dulbecco’s modified Eagle’s medium (DMEM; Gibco) supplemented with 5% hiFBS and 1% PSG. All cells were cultured at 37°C and 5% CO_2_.

For NK cell isolation, peripheral blood from healthy adult volunteers was collected in heparin tubes and the peripheral blood mononuclear cell fraction (PBMC) was obtained by density gradient centrifugation (Lymphoprep; Alere Technologies AS, Oslo). NK cells were purified from PBMCs by negative selection using a CD56^+^ NK cell isolation kit (Miltenyi Biotec, Germany). Isolated NK cells were cultured in RPMI supplemented with 10% hiFBS, 1% PSG, and 5 ng/ml recombinant human IL-15 (Biolegend). Before use, NK cells were rested overnight at a concentration of 1 × 10^6^ NK cells/ml at 37°C and 5% CO_2_.

Recombinant RSV-98-25147-X (RSV-A strain from the Netherlands, 1998, here referred to as RSV-X; GenBank FJ944820.1) encoding green fluorescent protein (GFP) was propagated in Vero cells [28]. Virus stock was purified between layers of 10% and 50% sucrose by ultracentrifugation. The 50% tissue culture infective dose (TCID50) per ml was determined on Vero cells using the Spearman and Karber method [29].

### RSV-specific multiplex immunoassay

To quantify the concentration of RSV-specific IgG and IgA, we performed a multiplex immunoassay following the previously described method [30]. Briefly, diluted serum samples were incubated with RSV post-fusion F-, pre-fusion F-, or nucleoprotein (N)-coupled beads. The captured antibodies were then detected using a secondary R-phycoerythrin-labeled F(abʹ)_2_ goat anti-human IgG, Fcγ fragment specific (Jackson ImmunoResearch Laboratories) or R-Phycoerythrin F(abʹ)_2_ goat anti-human IgA (SouthernBiotech).

For IgG subclass quantification, we used mouse anti-human IgG1 (Invitrogen | Thermo Fisher Scientific) and mouse anti-human IgG3 Hinge (Invitrogen | Thermo Fisher Scientific) as secondary monoclonal antibodies, followed by detection using R-Phycoerythrin F(abʹ)_2_ goat anti-mouse IgG, Fcγ fragment specific (Jackson ImmunoResearch laboratories).

Measurement of the samples was performed using a Flexmap 3D (Luminex Corp.) in combination with xPONENT 4.3 (DiaSorin/Luminex). Serum antibody concentrations were quantified in arbitrary units/mL (AU/ml) by interpolation from a five-parameter logistic curve of an in-house reference serum, using Bio-Plex Manager software version 6.2 (Bio-Rad Laboratories, Hercules, CA, USA).

### Virus neutralization (VN) assay

The virus neutralization assay was performed as described previously with a few adaptations [28]. Vero cells were seeded in 96-well plates one day prior to the neutralization assay. Heat-inactivated sera were diluted in 3-fold serial dilutions starting at 1:10 in DMEM supplemented with 2% hiFBS and 1% PSG. Serum dilutions, or a mock control with no serum, were mixed with an equal volume of RSV-X-GFP and incubated for 1 h at 37°C. Serum-virus mixtures were added to the Vero cell monolayers in triplicate (50 μl/well) and incubated for 2 h. Supernatant was removed and cells were overlayed with 1% methylcellulose in DMEM with 1% hiFBS and PSG. After two days of incubation, plaques were detected and counted with a fluorescence EliSpot reader (AID Autoimmun Diagnostika GmbH, Germany). Fifty percent plaque reduction neutralization titers (PRNT50) were calculated by nonlinear regression analysis with PRISM (Graphpad), normalized to control wells incubated with RSV without serum. Virus neutralization titers were only measured in sera of the Immfact study due to limitations in available sample volume for the OKIDOKI studies.

### Antibody-dependent NK cell activation (ADNKA) assay

Sterile ELISA plates (Immulon, Thermo Scientific) were coated with 0.5 μg/ml recombinant post-F (strain A2, 11049-V08B, Sino Biological) in 100 μl PBS, or coated without antigen as negative control, and incubated overnight at 4°C. Subsequently, wells were blocked with 5% BSA. After washing with PBS, 50 μl heat-inactivated serum (diluted 500× in PBS) was added to the wells and incubated for 2 h at 37°C. After incubation, 25 000 primary isolated NK cells in RPMI (10% hiFBS, 1% PSG) were added per well, in combination with brefeldin A (1:1000; BD Biosciences, New Jersey) and anti-human CD107a-PerCP/Cy5.5 (clone H4A3; Biolegend). This was incubated for 4 h at 37°C. After washing with FACS buffer, cells were stained for flow cytometric analysis. Extracellular staining was performed with anti-human CD3-FITC (clone UCHT1; Biolegend), anti-human CD56-PE (clone HCD56; Biolegend), and fixable viability staining-eFluor780 (Thermo Fisher Scientific). After fixing and permeabilization (fixation/permeabilization kit BD Biosciences), cells were stained intracellularly with anti-human IFN-γ-PE/Cy7 (clone B27; Biolegend). Cells were resuspended in FACS buffer and acquired on a FACS LSRFortessa X20 (BD Biosciences). Data was analyzed with FlowJo software. NK cells were gated as CD3^-^CD56^+^ cells. The gating strategy is depicted in Supplementary Fig. S1A. For each serum sample, results are expressed as the average percentage of CD107a + or IFN-γ+ NK cells from three different donors.

### Antibody-dependent cellular phagocytosis (ADCP) assay

A bead-based antibody-dependent phagocytosis assay was adapted from Ackerman *et al*. [31]. Recombinant post-F (Flys-GCN4) produced in eukaryotic cells [32] was biotinylated using a Sulfo-NHS-SS-Biotinylation kit according to manufacturer’s instructions (Thermo Fisher Scientific). Biotinylated post-F was coupled to 1.0 μm red fluorescent NeutrAvidin beads (Thermo Fisher Scientific) in a ratio of 1 μg protein to 1 μL beads and incubated overnight on a shaker at 4°C in the dark. Subsequently, beads were washed and blocked with 2% BSA for 1 h at room temperature. The antigen-coupled beads were then resuspended in PBS with 0.1% BSA to a concentration of 2.5 × 10^7^ beads/ml. Using a volume of 20 μl, 0.5 × 10^6^ beads were added per well of a 96-well plate. Heat-inactivated serum, 1250× diluted in PBS, was added 1:1 (v:v) to the antigen-coupled beads to a final dilution of 2500× and incubated for 2 h at 37°C. BSA-blocked beads without antigen with positive control serum added were used as negative control. After washing the beads twice with PBS supplemented with 0.1% BSA, 25 000 THP-1 cells in 100 μl RPMI (10% hiFBS and 1% PSG) were added per well. Cells were incubated with the serum-bead complexes for 1 h at 37°C. After washing with FACS buffer, cells were stained for flow cytometric analysis with fixable viability staining-eFluor780 (eBioscience, San Diego). Cells were fixed in 1% formaldehyde and acquired on a FACS LSRFortessa X20 (BD Bioscience). Data was analyzed with FlowJo software. The gating strategy is depicted in Supplementary Fig. S1B. Data are reported as phagocytic scores (iMFI), calculated as the percentage of bead-positive cells times the mean fluorescence intensity (MFI) of bead-positive cells divided by 1000. Data per sample are averaged from technical duplicates.

### Antibody-dependent complement deposition (ADCD) assay

A bead-based antibody-dependent complement deposition assay was adapted from Fischinger *et al*. [33]. Biotinylated post-F, as described for ADCP, was coupled to red fluorescent NeutrAvidin beads and beads were brought to a concentration of 2.5 × 10^7^ beads/ml as described above. 0.5 × 10^6^ beads were added per well to a 96-well plate. Heat-inactivated serum was added 1:1 (v:v) to the beads to a final dilution of 200× and incubated for 2 h at 37°C. BSA-blocked beads without antigen with positive control serum added were used as negative control. After washing with PBS with 0.1% BSA, 100 μL guinea pig complement (CL4051, Cedarlane laboratories, Burlington, Canada) diluted 1:25 in HBSS was added to the beads. After 15 min incubation at 37°C, beads were washed with cold FACS buffer (PBS supplemented with 2 mM EDTA and 0.5% BSA) and stained with anti-guinea pig complement C3-FITC (MP Biomedicals, Santa Ana) for 30 min. Samples were fixed in 1% formaldehyde and acquired on a FACS LSRFortessa X20 (BD Bioscience). The geometric mean fluorescence intensity (gMFI) of single beads was acquired with FlowJo software. The gating strategy is depicted in Supplementary Fig. S1C. Data per sample are averaged from technical duplicates.

### Optimization of Fc-effector function assays

Setting up the ADNKA, ADCP, and ADCD assays required optimization of a variety of assay conditions including antigen concentration, cell numbers, incubation time, and serum dilution. For example, the optimal serum dilution for each assay was determined by performing a serial dilution range using pooled serum of three healthy adult donors (Supplementary Fig. S2).

### Statistical analysis

Statistics were performed with GraphPad Prism version 9.3. To assess differences between age groups, a non-parametric Kruskal–Wallis test with Dunn’s multiple comparisons test was performed. Correlations were assessed using the non-parametric Spearman method.

For the longitudinal data, a non-parametric Mann–Whitney test was performed to assess differences between controls and the 2–3 months post-infection timepoint. A non–parametric Friedman test was performed to assess statistical differences over the entire follow-up period. To compare decay kinetics between antibody functions, data was normalized to 2–3 months post-infection. Subsequently, a two-way ANOVA with Tukey’s multiple comparison was performed to determine significant differences for each timepoint between each function.

## Results

### RSV seropositivity increases in the first years of life

**Figure 1.**
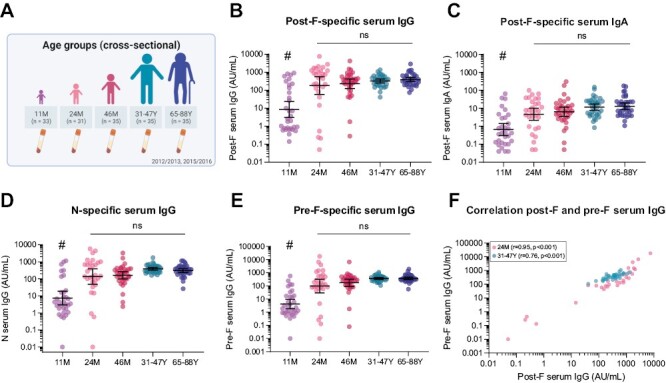
RSV-specific serum antibody levels in different age groups. RSV-specific serum IgG and IgA levels were measured with a multiplex immunoassay in 11-month (*n* = 32), 24-month (*n* = 30), and 46-month-old children (*n* = 35), adults (*n* = 35), and older adults (*n* = 35). (**A**) Schematic overview of the cross-sectional clinical study cohort used to assess differences between age groups. (**B**) Post-F-specific IgG levels. (**C**) Post-F-specific IgA levels. (**D**) N-specific IgG levels. (**E**) Pre-F-specific IgG levels. (**F**) Correlation of pre-F- and post-F-specific IgG levels for 24-month-old children (light pink) and adults (teal). All data points represent individual participants, and geometric mean concentrations (GMCs) with 95% confidence intervals are depicted. Data is analyzed by Kruskal–Wallis test and correlations are assessed using the Spearman method. # indicates statistical significance of at least *P* < 0.01 compared to all other age groups. AU/ml, arbitrary units per milliliter; ns, not significant.

To obtain a basic insight into the RSV antibody landscape in our study population (Fig. 1A), we assessed RSV-specific serum IgG and IgA levels using a bead-based multiplex immunoassay. In the 11-month-old group, approximately 53% (17/32) of the children were seropositive for RSV, based on post-F-specific IgG (Fig. 1B) and IgA (Fig. 1C) concentrations above a previously applied combined assay cut-off value of 1 AU/ml and 0.2 AU/ml, respectively [34]. As maternal antibodies have largely waned at the age of 11 months and IgA does not transfer across the placenta, these children have likely experienced a primary RSV infection [11]. At 24 months of age, seropositivity increased to 77% (23/30) and further increased to 94% (33/35) at 46 months of age. In the adult and older adult groups, all participants were seropositive for RSV. N- and pre-F-specific IgG levels showed a similar pattern (Fig. 1D–E), and pre-F-specific IgG levels correlated strongly with post-F-specific IgG levels (Fig. 1F). The numbers of seropositive individuals per age group correspond well with results from previous Dutch population studies [11, 34].

In line with the observed seropositivity, the geometric mean concentration (GMC) of post-F-, pre-F, and N-specific IgG antibodies and post-F-specific IgA antibodies in the 11-month-old group was significantly lower (Kruskal–Wallis, *P* < 0.01) than the GMCs observed in the other age groups (Fig. 1B–E). In addition, antibody levels were highly variable in the 11-month-old group. No significant difference in GMCs was observed between the 24- and 46-month-old, adult, and older adult groups (Kruskal–Wallis test), although there was an apparent decrease in the variability of antibody levels from early childhood to adulthood. Compared to that observed for RSV-specific IgG levels, post-F-specific IgA levels appear to show a slightly higher variability within age groups.

**Figure 2.**
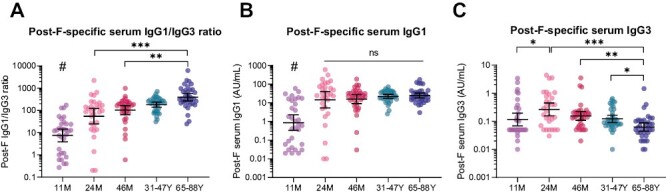
RSV-specific serum IgG subclasses in different age groups. Post-F-specific serum IgG subclass levels were measured with a multiplex immunoassay in 11-month (*n* = 32), 24-month (*n* = 30), and 46-month-old children (*n* = 35), adults (*n* = 35), and older adults (*n* = 35). (**A**) Post-F-specific IgG1/IgG3 ratio. (**B**) Post-F-specific IgG1 levels. (**C**) Post-F-specific IgG3 levels. All data points represent individual participants, and geometric mean concentrations (GMCs) with 95% confidence intervals are depicted. Data is analyzed by Kruskal–Wallis test. # indicates statistical significance of at least *P* < 0.01 compared to all other age groups. *P < 0.05; **P < 0.01; ***P < 0.001; ns, not significant. AU/ml, arbitrary units per milliliter.

As antibody subclass is an important determinant of Fc-mediated effector functionality [24], we also assessed post-F-specific IgG1 and IgG3 levels. IgG1 and IgG3 are more potent in inducing Fc-mediated effector functions than IgG2 and IgG4, where IgG3 is especially known to mediate ADNKA via enhanced binding to FcγRIII [35]. We found that older adults have a significantly higher IgG1/IgG3 ratio than children (Fig. 2A), which appears to be primarily due to a difference in IgG3 concentration. Whereas mean IgG1 levels did not differ between the age groups from 24 months of age onward (Fig. 2B), mean IgG3 levels decreased significantly as people age (Fig. 2C).

### Antibody-dependent NK cell activation is lower in children compared to adults

**Figure 3.**
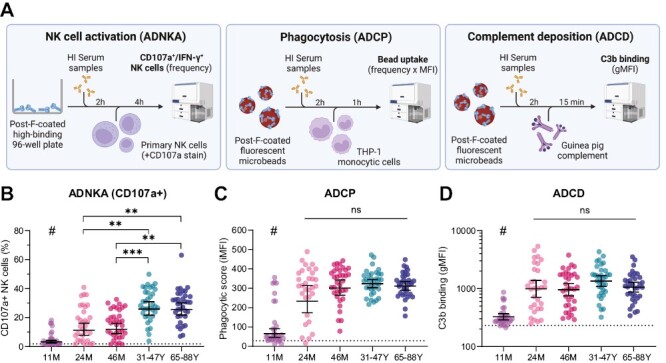
Fc-mediated antibody effector functions in different age groups. (**A**) Schematic overview of the methods used to assess ADNKA, ADCP, and ADCD in serum from 11-month (*n* = 33), 24-month (*n* = 31), and 46-month-old children (*n* = 35), adults (*n* = 35), and older adults (*n* = 35). (**B**) ADNKA with percentage of CD107a + NK cells as read-out; the average of three healthy NK cell donors is depicted for each participant. (**C**) ADCP by THP-1 cells with phagocytic score as read-out; data for each participant consists of the average of technical duplicates. (**D**) ADCD with gMFI as read-out; data for each participant consists of the average of technical duplicates. The dotted lines indicate the level of the negative control. All data points represent individual participants and geometric means with 95% confidence intervals are depicted. Data is analyzed by Kruskal–Wallis test. # indicates statistical significance of at least *P* < 0.01 compared to all other age groups. **P < 0.01; ***P < 0.001; ns, not significant. ADCD: antibody-dependent complement deposition; ADCP: antibody-dependent cellular phagocytosis; ADNKA: antibody-dependent NK cell activation; gMFI: geometric mean fluorescence intensity; HI: heat-inactivated; iMFI, integrated mean fluorescence intensity; NK cells: natural killer cells.

Next, we assessed the capacity of RSV post-F-specific serum antibodies to mediate ADNKA, ADCP by monocytes, and ADCD (Fig. 3A). Even though approximately half of the 11-month-old children were seropositive for RSV, the geometric mean percentage of activated NK cells, based on CD107a surface expression, was only slightly elevated above the negative control value (Fig. 3B). In addition, the 24- and 46-month-old children displayed significantly lower NK cell activation compared to adults and older adults (*P* < 0.01, Kruskal–Wallis test), despite having comparable GMCs for post-F-specific IgG. In contrast, no statistically significant difference in ADNKA was observed between adults and older adults. Interestingly, we did observe considerable within-group variation in ADNKA, even in adults and older adults where post-F-specific IgG levels showed only minor variation. Assessing ADNKA based on intracellular IFN-γ expression yielded highly similar results (Supplementary Fig. S3A).

Comparable to ADNKA, the 11-month-old group displayed only minor post-F-specific ADCP and ADCD (Fig. 3C and D). Although there was no statistically significant difference between 24- and 46-month-old children, adults, and older adults in the induction of ADCP and ADCD (Kruskal–Wallis test), there did appear to be a slight trend toward lower functionality in the younger age groups compared to adults. Similar to ADNKA, considerable within-group variation could be observed for ADCP and ADCD, which was especially pronounced in the 24-month-old group for ADCP.

### Lower antibody-dependent NK cell activation in 24- and 46-month-old children compared to adults is not explained by antibody levels

**Figure 4.**
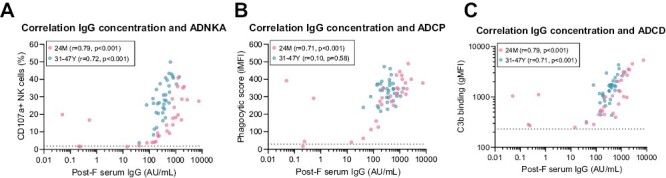
Correlation analysis between serum IgG levels and antibody functionalities for 24-month-old children and adults. Post-F-specific serum IgG levels and ADNKA, ADCP, and ADCD were determined for the 24-month-old group (light pink, *n* = 30) and adults (teal, *n* = 35). Correlations are shown between post-F serum IgG levels and ADNKA measured as percentage CD107a + NK cells (**A**), ADCP by THP-1 cells (**B**), and ADCD (**C**). Dotted lines indicate negative control (ADNKA, ADCP, ADCD). All data points represent individual participants. Correlations are assessed using the Spearman method. ADCD, antibody-dependent complement deposition; ADCP, antibody-dependent cellular phagocytosis; ADNKA, antibody-dependent NK cell activation; AU/mL, arbitrary units per milliliter; gMFI, geometric mean fluorescence intensity; iMFI, integrated mean fluorescence intensity; NK cells, natural killer cells.

The ADNKA assay revealed that serum from 24- and 46-month-old children induces significantly lower NK cell activation compared to serum obtained from adults and older adults while containing similar levels of post-F-specific IgG. To assess whether the lower capacity to mediate antibody-dependent NK cell activation of these sera is indeed not explained by lower antigen-specific IgG levels, we performed correlation analyses. Fig. 4A shows that there is a statistically significant positive correlation (Spearman) between post-F-specific IgG levels and antibody-dependent NK cell activation for both 24-month-old children (*r* = 0.79, *P* < 0.001) and adults (*r* = 0.72, *P* < 0.001). However, for the adult samples there is a clear skewing visible towards increased ADNKA compared to 24-month-old children, which strongly suggests that serum antibodies present in children indeed have a lower capacity to mediate ADNKA despite similar concentrations. For ADCP there is a significant correlation with post-F-specific IgG levels in 24-month-old children (*r* = 0.71, *P* < 0.001) but not adults (Fig. 4B), while the levels of ADCD correlate with post-F-specific IgG levels both in 24-month-old children (*r* = 0.79, *P* < 0.001) and adults (*r* = 0.71, *P* < 0.001; Fig. 4C). Notably, separation between 24-month-old children and adults as observed for ADNKA is much less pronounced for ADCP and ADCD. In 11- and 46-month-old children and older adults, ADNKA, ADCP, and ADCD all show a moderate to strong significant positive correlation with post-F-specific IgG levels (Supplementary Fig. S4). Of note, no clear separation can be observed for any of these functionalities between adults and older adults.

**Figure 5.**
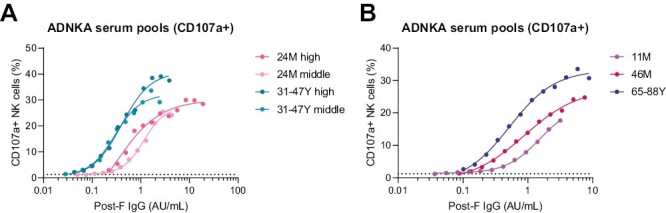
ADNKA titration curves of serum pools from different age groups. Serum pools were prepared from the 10 individuals with the highest post-F-specific IgG concentration for each age group (“high”) as well as pools of the 10 (adults) or 8 (24M) individuals with post-F-specific IgG levels around the GMC (“middle”). Post-F-specific IgG levels for each pool were measured with a multiplex immunoassay and ADNKA was assessed on a serial dilution range of each pool. (**A**) ADNKA titration of 24-month-old “high” (pink), 24-month-old “middle” (light pink), adults “high” (teal), and adults “middle” (light teal) serum pools, with percentage of CD107a + NK cells as read-out. (**B**) ADNKA titration of 11-month-old “high” (purple), 46-month-old “high” (dark pink), and older adults “high” (blue) serum pools, with percentage of CD107a + NK cells as read-out. The average of three healthy NK cell donors is depicted. The curves are fitted based on a 4-parameter nonlinear regression model. The dotted lines indicate the level of the negative control. ADNKA: antibody-dependent NK cell activation; AU/ml: arbitrary units per milliliter; GMC: geometric mean concentration; NK cells: natural killer cells.

To confirm the observed difference between antibodies from children and adults in ADNKA using a different approach, we prepared serum pools from the 10 individuals with the highest post-F-specific IgG levels in each age group (“high”). Additionally, for adults and 24-month-old children, we included a serum pool consisting of 10 (adults) or 8 (24M) individuals with post-F-specific IgG levels around the GMC (“middle”). The post-F-specific IgG levels of these pools were measured with the multiplex immunoassay, to determine their exact concentrations. Subsequently, a serial dilution range was made for each pool to measure ADNKA. Fig. 5A clearly shows that at the same concentrations both the “high” and “middle” curves from the 24-month-old children are shifted to the right compared to both of the adult curves, supporting our previous result that on average post-F-specific antibodies from 24-month-old children are weaker inducers of ADNKA than those from adults. A pattern consistent with our previous findings is also seen for the other age groups (Fig. 5B). ADNKA based on IFN-γ levels showed the same pattern (Supplementary Fig. S3B–C).

### Antibody concentration and functionality show the sharpest decline within the first 9 months post-infection in (older) adults

**Figure 6.**
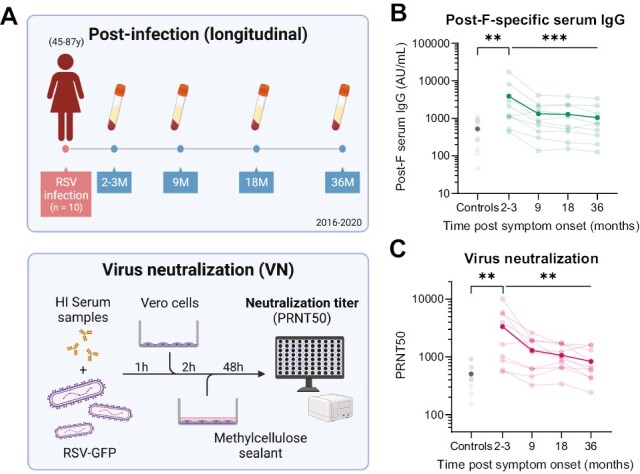
Serum antibody and neutralization titer kinetics post RSV infection. (**A**) Upper panel: schematic overview of the longitudinal clinical study cohort to assess antibody kinetics 2–36 months post-RSV infection in (older) adults (47–87 years old, *n* = 10). Lower panel: schematic representation of workflow for virus neutralization assay. (**B**) Post-F-specific serum IgG concentrations measured with a multiplex immunoassay in controls (*n* = 10) and convalescent individuals following RSV infection over time. (**C**) Virus neutralization titers in controls and convalescent individuals following RSV infection over time. Each line represents a unique individual, dark lines indicate the geometric mean of all participants. Light grey dots represent individual age-matched controls, dark grey dots represent the geometric mean level of the control samples. Differences between controls and 2–3 months post-symptom onset was assessed with a Mann–Whitney test. Differences over the complete follow-up time post-infection were assessed with a Friedman test. **P < 0.01; ***P < 0.001. AU/ml, arbitrary units per milliliter; PRNT50, 50% plaque reduction neutralization test.

Repeated infections with RSV throughout life, in the absence of substantial antigenic change, indicate that protective immunity from this respiratory virus is short-lived. It has previously been shown using a human challenge model for RSV that, following an initial boost, serum-neutralizing antibody titers wane substantially within the first 6 months after infection [19]. However, little is known about the durability of Fc-mediated antibody effector functionality following RSV infection. For this reason, we investigated the dynamics of RSV-specific antibody quantity and functionality in a longitudinal cohort of (older) adults up to three years post-infection (Fig. 6A). From 10 individuals (45–87 years old) with PCR-confirmed RSV infection, serum was collected at 2-3, 9, 18, and 36 months post-diagnosis. In addition to measuring IgG and IgA binding titers and Fc-functionality, virus neutralization was assessed for these samples (Fig. 6A).

As can be seen in Fig. 6B, the GMC of post-F-specific serum IgG at 2–3 months post-confirmed RSV infection is significantly elevated compared to that of controls (*P* < 0.01, Mann–Whitney test), supporting the notion that existing antibody levels can be boosted by natural infection, even in adults who have likely already experienced multiple infections throughout life. Over the course of 3 years, the GMCs of post-F-specific serum IgG decline (*P* < 0.001, Friedman test), with the sharpest decrease observed within the first 9 months post infection, after which the GMCs remain relatively stable. A similar pattern is observed for RSV N-specific IgG (Supplementary Fig. S5A) and post-F- and N-specific IgA (Supplementary Fig. S5B–C). Notably, the increase in IgA levels 2–3 months post-infection is not as pronounced as for IgG levels, which can be explained by the fact that IgA has a shorter half-life [36] and levels may therefore have already decreased by this time. Virus neutralization shows a similar pattern as IgG levels, with an elevated titer compared to controls at 2–3 months post-infection (*P* < 0.01) and the sharpest decrease in titer within the first 9 months post-infection (Fig. 6C).

**Figure 7.**
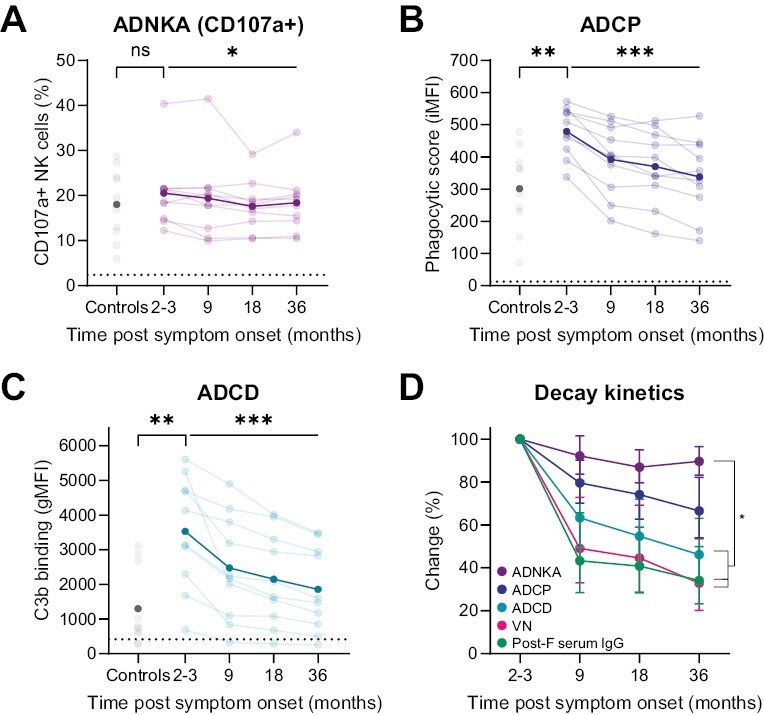
Fc-mediated antibody effector function kinetics post RSV infection. Serum Fc-functionality was assessed in longitudinal samples taken from (older) adults (*n* = 10) at 2–3, 9, 18, and 36 months post-RSV infection and from age-matched healthy controls (*n* = 10). Shown are antibody functions of samples for each timepoint, as indicated, assessed by (**A**) ADNKA with percentage of CD107a + NK cells as read-out, the average of three healthy NK cell donors is depicted for each participant; (**B**) ADCP by THP-1 cells with phagocytic score as read-out, data for each participant consists of the average of technical duplicates; (**C**) ADCD with geometric mean fluorescent intensity as read-out, data for each participant consists of the average of technical duplicates. The dotted lines indicate the level of the negative control. Each line represents a unique individual, dark lines indicate the geometric mean of all participants. Light grey dots represent individual age-matched controls, dark grey dots represent the geometric mean level of the control samples. Differences between controls and 2-3 months post-symptom onset was assessed with a Mann-Whitney test. Differences over the complete follow-up time post-infection were assessed with a Friedman test. (**D**) Overview of decay kinetics for antibody concentration and functionality normalized to 2-3 months post-infection with 95% confidence interval. A two-way ANOVA with Tukey’s multiple comparisons test was used to assess statistical significance of the difference in kinetics between antibody functions. Significance is indicated when there was a statistically significant difference for each timepoint between the two compared functions. *P < 0.05; **P < 0.01; ***P < 0.001; ns, not significant. ADNKA, antibody-dependent NK cell activation; ADCP, antibody-dependent cellular phagocytosis; ADCD: antibody-dependent complement deposition; gMFI: geometric mean fluorescence intensity; iMFI: integrated mean fluorescence intensity; NK cells: natural killer cells; VN: virus neutralization.

We subsequently assayed the various Fc-mediated antibody effector functions in these serum samples. In contrast to antibody levels and neutralization, ADNKA was not elevated at 2–3 months post-infection compared to controls and remained at approximately the same level from 2–3 to 36 months post-infection (Fig. 7A and Supplementary Fig. S5D). This observation suggests that either ADNKA is not boosted upon re-infection in (older) adults, or that considerable waning of this activity has already occurred before the 2–3 month time-point. Of note, one individual showed exceptionally high levels of ADNKA which did decrease slightly between 9 and 18 months post infection. ADCP and ADCD again showed a familiar pattern with elevated levels at 2–3 months post-infection compared to controls (*P* < 0.01) and the sharpest decrease within the first 9 months post-infection (Fig. 7B–C). Finally, Fig. 7D depicts the geometric mean levels of post-F-specific IgG concentration and the different antibody functionalities over time normalized to the 2–3 month time-point, to provide an indication of their relative decay kinetics. From this, it is evident that antibody concentration and serum neutralization capacity show the steepest decrease over time, followed by ADCD and ADCP, while ADNKA hardly decreases in this time window.

## Discussion

RSV vaccines targeting populations as diverse as infants, children, pregnant women, and older adults are currently in (late-stage) clinical development or have recently been approved for marketing [8–10]. Furthermore, while most correlates of protection studies only focus on antibody neutralization and binding titers, it has recently been shown that Fc-mediated effector functions likely play an important role in protection against RSV infection in non-human primates and human adults [22, 23]. To date, potential differences in RSV-specific antibody effector functions between different target groups have not been described, whereas such knowledge is pivotal for the rational development and improvement of (risk group-specific) vaccines. In this study, we found that serum antibodies of 24- and 46-month-old children induce significantly lower ADNKA than those of (older) adults while exhibiting a similar serum IgG concentration and ADCP/ADCD capacity. Moreover, our longitudinal data indicated that serum IgG levels and antibody functionality in (older) adults can be boosted, but show a sharp decline within the first 9 months and then remain relatively stable up to 3 years post infection, except for ADNKA which remains stable from 2-3 months up to 3 years post infection.

An inherent limitation to functional assays for ADNKA, ADCP, and ADCD is the fact that they generally exhibit a relatively limited dynamic range, as can be observed in Supplementary Fig. S2. As measuring multiple serum dilutions per sample is often practically not feasible and pre-adjusting the concentration of the input material potentially introduces additional variability, the limited dynamic range demands careful selection of the optimal serum dilution for each assay. In this study, the post-F-specific IgG concentrations fortunately lie within a relatively narrow range for the majority of samples (except for those of the 11-month-old children which are considerably lower) allowing for the assessment of functionality at a single dilution per assay. Additionally, there is no apparent plateau visible in the correlation plots of antibody concentration and functionality and the ADNKA results of the serial dilutions of serum pools are in line with the individual data, again suggesting that the functional measurements indeed lie within the dynamic ranges of the assays. However, we cannot completely rule out the possibility that additional differences in functionality between groups can be uncovered at other serum dilutions.

Previous research on SARS-CoV-2 showed an increased capacity for ADCP in adults compared to children, while no differences in ADCC were found [37]. Whereas the ADCP assay was similar to the set-up we used, ADCC was determined as FcγRIIIa activation on a reporter cell line. Since these results contrast with our observations for RSV, it is important to note that these were *primary* SARS-CoV-2 infections in both adults and children. As RSV re-infections occur frequently throughout life, adults have been exposed more often than children [3] and a proportion of 4-year-old children are also likely to have been infected at least twice [38]. Although there might be inherent immunological differences between children and adults [39], the number of previous RSV infections is likely one of the most important determinants of differences in the RSV-specific antibody response between these groups. A previous study with infants who had their primary RSV infection <1 year of age, showed that RSV-specific serum ADCC capacity is increased upon secondary infection [40]. Whether multiple re-infections result in an even further increase in ADCC capacity was not investigated, but our data suggest that this might be the case during childhood.

Repeated antigen exposure affects antibody functionality not only by increasing antibody concentration but also through antibody maturation [41]. One of the antibody characteristics that is changed through maturation is avidity, the multivalent binding strength between antigen and antibody. RSV-specific antibody avidity is low after primary infection, but increases upon re-infection [42]. Antibodies with increased avidity are expected to be more effective in mediating antibody effector functions. However, such an effect would be expected for all effector functions, not limited to ADNKA alone, and therefore likely does not solely explain the observed increase in ADNKA in adults compared to children.

Another essential characteristic of antibodies is their subclass, with IgG1 and IgG3 being particularly adept in inducing Fc-mediated effector functions. Notably, IgG3 is the most potent inducer of ADCC but also has a relatively short half-life [43]. Several studies by Wagner *et al.*, show that primary RSV infections lead to the induction of both IgG1 and IgG3, while re-infections primarily enhance IgG1 and IgG2 responses [44, 45]. Consistent with these findings, our results and existing literature show that children have higher IgG3 levels compared to (older) adults, resulting in a lower IgG1/IgG3 ratio [46]. Thus, children possess a more optimal IgG subclass ratio for inducing ADCC than adults. Nevertheless, our data showed *higher* ADNKA in adults, indicating that other antibody characteristics might be more important in supporting this functionality at advanced age.

Differential glycosylation of the IgG Fc-tail is another antibody characteristic that is increasingly recognized for its role in the regulation of Fc-mediated effector functions, which affects the interaction of antibodies with Fc-receptors. For example, afucosylated antibodies have an increased capacity to bind to FcγRIII, thereby inducing more ADCC than fucosylated antibodies [47]. Galactosylation increases this effect and, in addition, increases complement C1q-binding and downstream complement activation [48]. In contrast, sialylation has a slightly decreasing effect on FcyR binding [48, 49]. Repeated antigen exposure may modify glycosylation patterns and thereby have an effect on antibody functionality [50]. Moreover, several studies show an age-related effect on total IgG glycosylation, especially galactosylation [51–53]. The percentage of fucosylated and agalactosylated antibodies decreases from childhood to adolescence and whereas fucosylation does not show a clear pattern, agalactosylated antibodies increase again during adulthood. Notably, there is much variability in glycosylation patterns between individuals and the age effect also differs between males and females [52, 53]. Differential glycosylation in children and adults is a possible explanation for our results, but more research on RSV-specific antibody glycosylation in different age groups is needed.

Finally, the position of the epitope to which antibodies bind affects Fc-mediated effector functions by influencing the positioning of the effector cell/molecule relative to the target cell. For example, antibodies binding to epitopes closer to the membrane are better inducers of ADCC and ADCD, while binding to epitopes more distal from the membrane are better inducers of ADCP [54]. However, because in our experimental set-up the antigen is bound to beads or plate in a random orientation, we expect no influence of epitope position in our assays.

In this study, we investigated antibody effector functions using post-F as an antigen. Other important RSV surface antigens are pre-F and the attachment protein G. It is currently unclear whether using a different RSV antigen would yield different results. In their study, Bartsch *et al*. found a lower Fc-mediated antibody functionality against the G protein compared to post-F [22]. However, it is unclear to what extent this finding relates to differences in antibody concentrations as it has previously been suggested that post-F and pre-F serum IgG have similar concentrations, whereas G-specific serum IgG has a considerably lower concentration [11]. Other studies investigating Fc-glycosylation patterns found differences within individuals between antibodies specific for two HIV antigens, one influenza antigen, and bulk antibodies [55] or between membrane-bound versus soluble antigens [56]. It is not known whether Fc-glycosylation patterns of antibodies targeting different RSV antigens differ as well. However, as post-F has many shared epitopes with pre-F and their IgG levels correlate strongly, we expect there will be no major differences in Fc-functionality between these antigens. As F-specific antibodies seem to be most abundantly present, these likely play the most important role in determining Fc-mediated effector functions during RSV infection.

Whereas many studies only assess the levels of serum IgG, the role of IgA during RSV infection cannot be neglected. Especially in the mucosa, IgA is essential for virus neutralization, and high nasal IgA titers are a better correlate of protection than serum IgG [19, 57]. Similar to IgG, IgA can mediate Fc-effector functions through binding to Fcα receptors present on e.g. monocytes, macrophages, and neutrophils [36]. Importantly, secreted IgA, mostly present in the mucosa, is less efficient in inducing Fc-mediated antibody effector functions than serum IgA [58]. Although most research on IgA effector functions concerns tumor immunology, it has previously been shown that SARS-CoV-2-, HIV-, and influenza virus-specific IgAs are able to activate neutrophils [58–62]. For RSV, it has been demonstrated *in vitro* that palivizumab-IgA induces lysis through ADCC by neutrophils better than palivizumab-IgG [63]. However, additional *in vivo* experiments with FcαRI transgenic mice showed that the IgA protection against RSV infection was independent of Fc-receptor interaction [63]. No other studies have looked at RSV-specific IgA in the context of Fc-mediated effector functions, so its role in RSV disease remains to be elucidated. Of note, however, the sera assessed in the current study contain RSV-specific IgA which potentially contributes to the observed effector functions.

The current study design does not allow for an investigation of correlates of protection for RSV. Previously however, ADCC was shown to exert protective effects in several viral infections, e.g. for SARS-CoV-2, HIV, and influenza virus [64–67], but may also worsen disease by an overactive immune response, as seen for influenza virus [68, 69]. During RSV infection, NK cells may help fight the infection through cytotoxicity and by attracting other immune cells. However, later during infection, NK cells may worsen disease by an excessive response, contributing to lung damage (reviewed in [70]). We previously showed that serum of infants with an acute severe respiratory viral infection (including RSV) induces less ADNKA compared to uninfected controls [71]. Additionally, Bartsch *et al*. show that Fc-mediated antibody effector functions, especially ADCP, but also ADCC, associated with protection from RSV infection after vaccination in adults [22]. These studies suggest that sufficient induction of ADCC is indeed important to decrease the severity of RSV disease. It should be noted that not only antibody-mediated mechanisms, but also RSV protein-specific memory T-cell responses have a protective role against the severity of RSV disease and viral load, as was shown in a controlled human infection model [72]. Our data do not reveal a difference between adults and older adults in functional antibody responses. The increased susceptibility of older adults to severe RSV disease may therefore be explained by deficits in other immune compartments, such as innate or T cells. Further research is necessary to define the relative role of various immune mechanisms in protection against RSV infection and disease.

Our longitudinal data showed that ADNKA remains stable from 2 to 3 months up to 3 years post-infection in adults. A possible explanation for this observation is that ADNKA capacity is not boosted in adults upon re-infection or, alternatively, is boosted but decreases again very rapidly, before the 2–3 months timepoint. In line with the latter explanation, Kaul *et al*. show that ADCC drops one month after primary RSV infection in infants <1 year of age [40] and Meguro *et al*. show that ADCC peaks already 10 days post-infection in infants, with a rapid decline over time [73]. In studies with other viruses, it is also shown that ADCC-inducing antibodies decline rapidly after infection or, with no knowledge of prior infection, remain stable over time. For SARS-CoV-2, a quick decrease in ADNKA is described between 1 and 4–8 months post-infection [74]. Pre-existing ADCC-mediating influenza antibodies remain stable over time for at least 6 months [75], similar to our observations for RSV.

Other longitudinal RSV studies have mainly focused on antibody concentrations and neutralization titers. Our data align well with previous research, in which participants were followed up to a year post-RSV infection. Blunck *et al*. found that RSV neutralization titers remain stable for at least half a year in uninfected individuals [76]. Furthermore, they showed that in acutely infected individuals neutralization titers increase, and then stay stable for at least 4 months. Multiple studies demonstrate that one month after RSV infection in adults serum neutralization titers have increased, but 6–12 months later they have decreased to almost baseline again [13, 19, 77]. A downside of our study design is the lack of a baseline sample and relatively late sampling of the first timepoints. For this reason, additional studies (e.g. controlled human infection models) are necessary to specifically unravel the boosting and decay of antibody Fc-mediated effector functions at early timepoints after infection.

In conclusion, we found that similar to antibody levels, the elevation of the functional RSV-specific antibody response is of limited duration, providing a possible explanation for frequent re-infections. Additionally, our study yielded novel insights into the differences between children and adults in functional antibody responses, which warrant further investigations to define their role in protection, taking into account potential differences between risk groups which might require a tailored approach to vaccination. Different vaccine adjuvants have previously been shown to steer the production of antibodies with different functionalities [78, 79]. While this provides opportunities for rational vaccine design, more research is needed to fully understand the factors underlying the regulation of antibody quality. A thorough understanding of antibody functionality in individual risk groups is essential for future vaccine strategies for both infants and older adults.

## Supplementary Material

uxad101_suppl_Supplementary_Figures

## Data Availability

The data underlying this article will be shared on reasonable request to the corresponding author.

## Abbreviations:

ADCD: antibody-dependent complement deposition
ADCP: antibody-dependent cellular phagocytosis
ADNKA: antibody-dependent NK cell activation
ADCC: antibody-dependent cellular cytotoxicity
AU: arbitrary units
BSA: bovine serum albumin
FBS: fetal bovine serum
GFP: green fluorescent protein
GMC: geometric mean concentration
gMFI: geometric mean fluorescence intensity
HI: heat inactivated
IFN: interferon
Ig: immunoglobulin
iMFI: integrated mean fluorescence intensity
NK: natural killer
PBMC: peripheral blood mononuclear cells
Post-F: postfusion F protein
Pre-F: prefusion F protein
PRNT50: 50% plaque reduction neutralization test
PSG: penicillin/streptomycin/glutamine
RSV: respiratory syncytial virus
VN: virus neutralization

## Clinical Trial Registration

OKIDOKI and ILI studies are registered at trialsearch.who.int: NTR3614, NTR5405, NTR3386. The Immfact study is registered at CCMO: NL46795.094.13.

## References

[1] Shi T , Denouel A, Tietjen AK, Campbell I, Moran E, Li X, et al. Global disease burden estimates of respiratory syncytial virus-associated acute respiratory infection in older adults in 2015: a systematic review and meta-analysis. J Infect Dis 2019, 222 (Suppl 7), S577–S583. 10.1093/infdis/jiz059 30880339

[2] Shi T , McAllister DA, O’Brien KL, Simoes EAF, Madhi SA, Gessner BD, et al.; RSV Global Epidemiology Network. Global, regional, and national disease burden estimates of acute lower respiratory infections due to respiratory syncytial virus in young children in 2015: a systematic review and modelling study. Lancet 2017, 390, 946–58. doi:10.1016/S0140-6736(17)30938-8. 28689664 PMC5592248

[3] Hall CB , Walsh EE, Long CE, Schnabel KC. Immunity to and frequency of reinfection with respiratory syncytial virus. J Infect Dis 1991, 163, 693–8. doi:10.1093/infdis/163.4.693. 2010624

[4] Henderson FW , Collier AM, Clyde WA Jr, Denny FW. Respiratory-syncytial-virus infections, reinfections and immunity. A prospective, longitudinal study in young children. N Engl J Med 1979, 300, 530–4. doi:10.1056/NEJM197903083001004. 763253

[5] Garegnani L , Styrmisdottir L, Roson Rodriguez P, Escobar Liquitay CM, Esteban I, Franco JV. Palivizumab for preventing severe respiratory syncytial virus (RSV) infection in children. Cochrane Database Syst Rev 2021, 11, CD013757. doi:10.1002/14651858.CD013757.pub2. 34783356 PMC8594174

[6] Hammitt LL , Dagan R, Yuan Y, Baca Cots M, Bosheva M, Madhi SA, et al.; MELODY Study Group. Nirsevimab for prevention of RSV in healthy late-preterm and term infants. N Engl J Med 2022, 386, 837–46. doi:10.1056/NEJMoa2110275. 35235726

[7] Muller WJ , Madhi SA, Seoane Nunez B, Baca Cots M, Bosheva M, Dagan R, et al. Nirsevimab for prevention of RSV in term and late-preterm infants. N Engl J Med 2023, 388, 1533–4. doi:10.1056/nejmc2214773. 37018470

[8] Mazur NI , Terstappen J, Baral R, Bardaji A, Beutels P, Buchholz UJ, et al. Respiratory syncytial virus prevention within reach: the vaccine and monoclonal antibody landscape. Lancet Infect Dis 2023, 23, e2–e21. doi:10.1016/S1473-3099(22)00291-2. 35952703 PMC9896921

[9] Kampmann B , Madhi SA, Munjal I, Simoes EAF, Pahud BA, Llapur C, et al. Bivalent prefusion F vaccine in pregnancy to prevent RSV illness in infants. N Engl J Med 2023, 388, 1451–1464. 37018474 10.1056/NEJMoa2216480

[10] Walsh EE , Perez Marc G, Zareba AM, Falsey AR, Jiang Q, Patton M, et al. Efficacy and safety of a bivalent RSV prefusion F vaccine in older adults. N Engl J Med 2023, 388, 1465–77. doi:10.1056/nejmoa2213836. 37018468

[11] Berbers G , Mollema L, van der Klis F, den Hartog G, Schepp R. Antibody responses to respiratory syncytial virus: a cross-sectional serosurveillance study in the dutch population focusing on infants younger than 2 years. J Infect Dis 2021, 224, 269–78. doi:10.1093/infdis/jiaa483. 32964923 PMC8280491

[12] Eick A , Karron R, Shaw J, Thumar B, Reid R, Santosham M, et al. The role of neutralizing antibodies in protection of American Indian infants against respiratory syncytial virus disease. Pediatr Infect Dis J 2008, 27, 207–12. doi:10.1097/INF.0b013e31815ac585. 18277934

[13] Ascough S , Dayananda P, Kalyan M, Kuong SU, Gardener Z, Bergstrom E, et al. Divergent age-related humoral correlates of protection against respiratory syncytial virus infection in older and young adults: a pilot, controlled, human infection challenge model. Lancet Healthy Longev 2022, 3, e405–16. doi:10.1016/S2666-7568(22)00103-9. 36098319

[14] Walsh EE , Peterson DR, Falsey AR. Risk factors for severe respiratory syncytial virus infection in elderly persons. J Infect Dis 2004, 189, 233–8. doi:10.1086/380907. 14722887

[15] Taleb SA , Al-Ansari K, Nasrallah GK, Elrayess MA, Al-Thani AA, Derrien-Colemyn A, et al. Level of maternal respiratory syncytial virus (RSV) F antibodies in hospitalized children and correlates of protection. Int J Infect Dis 2021, 109, 56–62. doi:10.1016/j.ijid.2021.06.015. 34118428

[16] Nyiro JU , Sande CJ, Mutunga M, Kiyuka PK, Munywoki PK, Scott JA, et al. Absence of association between cord specific antibody levels and severe respiratory syncytial virus (RSV) disease in early infants: a case control study from coastal Kenya. PLoS One 2016, 11, e0166706. doi:10.1371/journal.pone.0166706. 27851799 PMC5113039

[17] Chu HY , Tielsch J, Katz J, Magaret AS, Khatry S, LeClerq SC, et al. Transplacental transfer of maternal respiratory syncytial virus (RSV) antibody and protection against RSV disease in infants in rural Nepal. J Clin Virol 2017, 95, 90–5. doi:10.1016/j.jcv.2017.08.017. 28903080 PMC5625849

[18] Jans J , Wicht O, Widjaja I, Ahout IM, de Groot R, Guichelaar T, et al. Characteristics of RSV-specific maternal antibodies in plasma of hospitalized, acute RSV patients under three months of age. PLoS One 2017, 12, e0170877. doi:10.1371/journal.pone.0170877. 28135305 PMC5279754

[19] Habibi MS , Jozwik A, Makris S, Dunning J, Paras A, DeVincenzo JP, et al.; Mechanisms of Severe Acute Influenza Consortium Investigators. Impaired antibody-mediated protection and defective IgA B-cell memory in experimental infection of adults with respiratory syncytial virus. Am J Respir Crit Care Med 2015, 191, 1040–9. doi:10.1164/rccm.201412-2256OC. 25730467 PMC4435460

[20] Polack FP , Teng MN, Collins PL, Prince GA, Exner M, Regele H, et al. A role for immune complexes in enhanced respiratory syncytial virus disease. J Exp Med 2002, 196, 859–65. doi:10.1084/jem.20020781. 12235218 PMC2194058

[21] Openshaw PJ , Tregoning JS. Immune responses and disease enhancement during respiratory syncytial virus infection. Clin Microbiol Rev 2005, 18, 541–55. doi:10.1128/CMR.18.3.541-555.2005. 16020689 PMC1195968

[22] Bartsch YC , Cizmeci D, Kang J, Zohar T, Periasamy S, Mehta N, et al. Antibody effector functions are associated with protection from respiratory syncytial virus. Cell 2022, 185, 4873–4886.e10. doi:10.1016/j.cell.2022.11.012. 36513064

[23] Zohar T , Hsiao JC, Mehta N, Das J, Devadhasan A, Karpinski W, et al. Upper and lower respiratory tract correlates of protection against respiratory syncytial virus following vaccination of nonhuman primates. Cell Host Microbe 2022, 30, 41–52.e5. doi:10.1016/j.chom.2021.11.006. 34879230

[24] van Erp EA , Luytjes W, Ferwerda G, van Kasteren PB. Fc-mediated antibody effector functions during respiratory syncytial virus infection and disease. Front Immunol 2019, 10, 548. doi:10.3389/fimmu.2019.00548. 30967872 PMC6438959

[25] Bosch A , van Houten MA, Bruin JP, Wijmenga-Monsuur AJ, Trzcinski K, Bogaert D, et al. Nasopharyngeal carriage of Streptococcus pneumoniae and other bacteria in the 7th year after implementation of the pneumococcal conjugate vaccine in the Netherlands. Vaccine 2016, 34, 531–9. 26667610 10.1016/j.vaccine.2015.11.060

[26] van Beek J , Veenhoven RH, Bruin JP, van Boxtel RAJ, de Lange MMA, Meijer A, et al. Influenza-like illness incidence is not reduced by influenza vaccination in a cohort of older adults, despite effectively reducing laboratory-confirmed influenza virus infections. J Infect Dis 2017, 216, 415–24. doi:10.1093/infdis/jix268. 28931240 PMC7107403

[27] Kroes MM , van Vliet LC, Jacobi RHJ, Kuipers B, Pieren DKJ, Miranda-Bedate A, et al. Long lasting antibodies from convalescent pertussis patients induce ROS production and bacterial killing by human neutrophils. Front Cell Infect Microbiol 2022, 12, 888412. doi:10.3389/fcimb.2022.888412. 35646735 PMC9135168

[28] van Remmerden Y , Xu F, van Eldik M, Heldens JG, Huisman W, Widjojoatmodjo MN. An improved respiratory syncytial virus neutralization assay based on the detection of green fluorescent protein expression and automated plaque counting. Virol J 2012, 9, 253. doi:10.1186/1743-422X-9-253. 23114196 PMC3514128

[29] Hierholzer JC , Killington RA. Virus isolation and quantitation. In: Mahy BWJ, Kangro HO, editors. Virology Methods Manual. London, UK; San Diego, CA, USA: Academic Press; 1996. p. 24–32.

[30] Schepp RM , de Haan CAM, Wilkins D, Layman H, Graham BS, Esser MT, et al. Development and standardization of a high-throughput multiplex immunoassay for the simultaneous quantification of specific antibodies to five respiratory syncytial virus proteins. mSphere 2019, 4, e00236–19. doi:10.1128/mSphere.00236-19. 31019002 PMC6483049

[31] Ackerman ME , Moldt B, Wyatt RT, Dugast AS, McAndrew E, Tsoukas S, et al. A robust, high-throughput assay to determine the phagocytic activity of clinical antibody samples. J Immunol Methods 2011, 366, 8–19. doi:10.1016/j.jim.2010.12.016. 21192942 PMC3050993

[32] Widjaja I , Rigter A, Jacobino S, van Kuppeveld FJ, Leenhouts K, Palomo C, et al. Recombinant soluble respiratory syncytial virus f protein that lacks heptad repeat B, contains a GCN4 trimerization motif and is not cleaved displays prefusion-like characteristics. PLoS One 2015, 10, e0130829. doi:10.1371/journal.pone.0130829. 26107504 PMC4481108

[33] Fischinger S , Fallon JK, Michell AR, Broge T, Suscovich TJ, Streeck H, et al. A high-throughput, bead-based, antigen-specific assay to assess the ability of antibodies to induce complement activation. J Immunol Methods 2019, 473, 112630. doi:10.1016/j.jim.2019.07.002. 31301278 PMC6722412

[34] Andeweg SP , Schepp RM, van de Kassteele J, Mollema L, Berbers GAM, van Boven M. Population-based serology reveals risk factors for RSV infection in children younger than 5 years. Sci Rep 2021, 11, 8953. doi:10.1038/s41598-021-88524-w. 33903695 PMC8076290

[35] Bruhns P , Iannascoli B, England P, Mancardi DA, Fernandez N, Jorieux S, et al. Specificity and affinity of human Fcgamma receptors and their polymorphic variants for human IgG subclasses. Blood 2009, 113, 3716–25. doi:10.1182/blood-2008-09-179754. 19018092

[36] Davis SK , Selva KJ, Kent SJ, Chung AW. Serum IgA Fc effector functions in infectious disease and cancer. Immunol Cell Biol 2020, 98, 276–86. doi:10.1111/imcb.12306. 31785006 PMC7217208

[37] Pierce CA , Preston-Hurlburt P, Dai Y, Aschner CB, Cheshenko N, Galen B, et al. Immune responses to SARS-CoV-2 infection in hospitalized pediatric and adult patients. Sci Transl Med 2020, 12, eabd5487. doi:10.1126/scitranslmed.abd5487. 32958614 PMC7658796

[38] Kutsaya A , Teros-Jaakkola T, Kakkola L, Toivonen L, Peltola V, Waris M, et al. Prospective clinical and serological follow-up in early childhood reveals a high rate of subclinical RSV infection and a relatively high reinfection rate within the first 3 years of life. Epidemiol Infect 2016, 144, 1622–33. doi:10.1017/S0950268815003143. 26732801 PMC9150639

[39] Simon AK , Hollander GA, McMichael A. Evolution of the immune system in humans from infancy to old age. Proc Biol Sci 2015, 282, 20143085. doi:10.1098/rspb.2014.3085. 26702035 PMC4707740

[40] Kaul TN , Welliver RC, Ogra PL. Development of antibody-dependent cell-mediated cytotoxicity in the respiratory tract after natural infection with respiratory syncytial virus. Infect Immun 1982, 37, 492–8. doi:10.1128/iai.37.2.492-498.1982. 7118247 PMC347561

[41] Nielsen SCA , Roskin KM, Jackson KJL, Joshi SA, Nejad P, Lee JY, et al. Shaping of infant B cell receptor repertoires by environmental factors and infectious disease. Sci Transl Med 2019, 11, eaat2004. doi:10.1126/scitranslmed.aat2004. 30814336 PMC6733608

[42] Meurman O , Waris M, Hedman K. Immunoglobulin G antibody avidity in patients with respiratory syncytial virus infection. J Clin Microbiol 1992, 30, 1479–84. doi:10.1128/jcm.30.6.1479-1484.1992. 1624567 PMC265314

[43] Vidarsson G , Dekkers G, Rispens T. IgG subclasses and allotypes: from structure to effector functions. Front Immunol 2014, 5, 520. doi:10.3389/fimmu.2014.00520. 25368619 PMC4202688

[44] Wagner DK , Graham BS, Wright PF, Walsh EE, Kim HW, Reimer CB, et al. Serum immunoglobulin G antibody subclass responses to respiratory syncytial virus F and G glycoproteins after primary infection. J Clin Microbiol 1986, 24, 304–6. doi:10.1128/jcm.24.2.304-306.1986. 3755731 PMC268896

[45] Wagner DK , Muelenaer P, Henderson FW, Snyder MH, Reimer CB, Walsh EE, et al. Serum immunoglobulin G antibody subclass response to respiratory syncytial virus F and G glycoproteins after first, second, and third infections. J Clin Microbiol 1989, 27, 589–92. doi:10.1128/jcm.27.3.589-592.1989. 2715331 PMC267370

[46] Jounai N , Yoshioka M, Tozuka M, Inoue K, Oka T, Miyaji K, et al. Age-specific profiles of antibody responses against respiratory syncytial virus infection. EBioMedicine 2017, 16, 124–35. doi:10.1016/j.ebiom.2017.01.014. 28111238 PMC5474434

[47] Shields RL , Lai J, Keck R, O’Connell LY, Hong K, Meng YG, et al. Lack of fucose on human IgG1 N-linked oligosaccharide improves binding to human Fcgamma RIII and antibody-dependent cellular toxicity. J Biol Chem 2002, 277, 26733–40. doi:10.1074/jbc.M202069200. 11986321

[48] Dekkers G , Treffers L, Plomp R, Bentlage AEH, de Boer M, Koeleman CAM, et al. Decoding the human immunoglobulin G-glycan repertoire reveals a spectrum of Fc-receptor- and complement-mediated-effector activities. Front Immunol 2017, 8, 877. doi:10.3389/fimmu.2017.00877. 28824618 PMC5539844

[49] Kaneko Y , Nimmerjahn F, Ravetch JV. Anti-inflammatory activity of immunoglobulin G resulting from Fc sialylation. Science 2006, 313, 670–3. doi:10.1126/science.1129594. 16888140

[50] Alter G , Ottenhoff THM, Joosten SA. Antibody glycosylation in inflammation, disease and vaccination. Semin Immunol 2018, 39, 102–10. doi:10.1016/j.smim.2018.05.003. 29903548 PMC8731230

[51] Pucic M , Muzinic A, Novokmet M, Skledar M, Pivac N, Lauc G, et al. Changes in plasma and IgG N-glycome during childhood and adolescence. Glycobiology 2012, 22, 975–82. doi:10.1093/glycob/cws062. 22426998

[52] Chen G , Wang Y, Qiu L, Qin X, Liu H, Wang X, et al. Human IgG Fc-glycosylation profiling reveals associations with age, sex, female sex hormones and thyroid cancer. J Proteomics 2012, 75, 2824–34. doi:10.1016/j.jprot.2012.02.001. 22365975

[53] Kristic J , Vuckovic F, Menni C, Klaric L, Keser T, Beceheli I, et al. Glycans are a novel biomarker of chronological and biological ages. J Gerontol A Biol Sci Med Sci 2014, 69, 779–89. doi:10.1093/gerona/glt190. 24325898 PMC4049143

[54] Cleary KLS , Chan HTC, James S, Glennie MJ, Cragg MS. Antibody distance from the cell membrane regulates antibody effector mechanisms. J Immunol 2017, 198, 3999–4011. doi:10.4049/jimmunol.1601473. 28404636 PMC5424080

[55] Mahan AE , Jennewein MF, Suscovich T, Dionne K, Tedesco J, Chung AW, et al. Antigen-specific antibody glycosylation is regulated via vaccination. PLoS Pathog 2016, 12, e1005456. doi:10.1371/journal.ppat.1005456. 26982805 PMC4794126

[56] Larsen MD , de Graaf EL, Sonneveld ME, Plomp HR, Nouta J, Hoepel W, et al.; Amsterdam UMC COVID-19. Afucosylated IgG characterizes enveloped viral responses and correlates with COVID-19 severity. Science 2021, 371, eabc8378. doi:10.1126/science.abc8378. 33361116 PMC7919849

[57] Habibi MS , Thwaites RS, Chang M, Jozwik A, Paras A, Kirsebom F, et al. Neutrophilic inflammation in the respiratory mucosa predisposes to RSV infection. Science 2020, 370, eaba9301. doi:10.1126/science.aba9301. 33033192 PMC7613218

[58] Stacey HD , Golubeva D, Posca A, Ang JC, Novakowski KE, Zahoor MA, et al. IgA potentiates NETosis in response to viral infection. Proc Natl Acad Sci U S A 2021, 118, e2101497118. doi:10.1073/pnas.2101497118. 34183391 PMC8271757

[59] Duchemin M , Tudor D, Cottignies-Calamarte A, Bomsel M. Antibody-dependent cellular phagocytosis of HIV-1-infected cells is efficiently triggered by IgA targeting HIV-1 envelope subunit gp41. Front Immunol 2020, 11, 1141. doi:10.3389/fimmu.2020.01141. 32582208 PMC7296124

[60] Freyn AW , Han J, Guthmiller JJ, Bailey MJ, Neu K, Turner HL, et al. Influenza hemagglutinin-specific IgA Fc-effector functionality is restricted to stalk epitopes. Proc Natl Acad Sci U S A 2021, 118, e2018102118. doi:10.1073/pnas.2018102118. 33593910 PMC7923359

[61] Mullarkey CE , Bailey MJ, Golubeva DA, Tan GS, Nachbagauer R, He W, et al. Broadly neutralizing hemagglutinin stalk-specific antibodies induce potent phagocytosis of immune complexes by neutrophils in an Fc-dependent manner. mBio 2016, 7, e01624–16. doi:10.1128/mBio.01624-16. 27703076 PMC5050345

[62] Duchemin M , Khamassi M, Xu L, Tudor D, Bomsel M. IgA targeting human immunodeficiency virus-1 envelope gp41 triggers antibody-dependent cellular cytotoxicity cross-clade and cooperates with gp41-specific IgG to increase cell lysis. Front Immunol 2018, 9, 244. doi:10.3389/fimmu.2018.00244. 29651286 PMC5884934

[63] Jacobino SR , Nederend M, Reijneveld JF, Augustijn D, Jansen JHM, Meeldijk J, et al. Reformatting palivizumab and motavizumab from IgG to human IgA impairs their efficacy against RSV infection in vitro and in vivo. MAbs 2018, 10, 453–62. doi:10.1080/19420862.2018.1433974. 29553863 PMC5939987

[64] DiLillo DJ , Palese P, Wilson PC, Ravetch JV. Broadly neutralizing anti-influenza antibodies require Fc receptor engagement for in vivo protection. J Clin Invest 2016, 126, 605–10. doi:10.1172/JCI84428. 26731473 PMC4731186

[65] Jegaskanda S , Luke C, Hickman HD, Sangster MY, Wieland-Alter WF, McBride JM, et al. Generation and protective ability of influenza virus-specific antibody-dependent cellular cytotoxicity in humans elicited by vaccination, natural infection, and experimental challenge. J Infect Dis 2016, 214, 945–52. doi:10.1093/infdis/jiw262. 27354365 PMC4996149

[66] Wren LH , Chung AW, Isitman G, Kelleher AD, Parsons MS, Amin J, et al.; ADCC study collaboration investigators. Specific antibody-dependent cellular cytotoxicity responses associated with slow progression of HIV infection. Immunology 2013, 138, 116–23. doi:10.1111/imm.12016. 23173935 PMC3575764

[67] Fuentes-Villalobos F , Garrido JL, Medina MA, Zambrano N, Ross N, Bravo F, et al.; COVID-19 South Chile Group. Sustained antibody-dependent NK cell functions in mild COVID-19 outpatients during convalescence. Front Immunol 2022, 13, 796481. doi:10.3389/fimmu.2022.796481. 35197972 PMC8859986

[68] Ye ZW , Yuan S, Poon KM, Wen L, Yang D, Sun Z, et al. Antibody-dependent cell-mediated cytotoxicity epitopes on the hemagglutinin head region of pandemic H1N1 influenza virus play detrimental roles in H1N1-infected mice. Front Immunol 2017, 8, 317. doi:10.3389/fimmu.2017.00317. 28377769 PMC5359280

[69] Co MD , Terajima M, Thomas SJ, Jarman RG, Rungrojcharoenkit K, Fernandez S, et al. Relationship of preexisting influenza hemagglutination inhibition, complement-dependent lytic, and antibody-dependent cellular cytotoxicity antibodies to the development of clinical illness in a prospective study of A(H1N1)pdm09 Influenza in children. Viral Immunol 2014, 27, 375–82. doi:10.1089/vim.2014.0061. 25141276 PMC4183906

[70] Bhat R , Farrag MA, Almajhdi FN. Double-edged role of natural killer cells during RSV infection. Int Rev Immunol 2020, 39, 233–44. doi:10.1080/08830185.2020.1770748. 32469615

[71] van Erp EA , Lakerveld AJ, de Graaf E, Larsen MD, Schepp RM, Hipgrave Ederveen AL, et al. Natural killer cell activation by respiratory syncytial virus-specific antibodies is decreased in infants with severe respiratory infections and correlates with Fc-glycosylation. Clin Transl Immunol 2020, 9, e1112. doi:10.1002/cti2.1112. PMC7029726 32099650

[72] Jozwik A , Habibi MS, Paras A, Zhu J, Guvenel A, Dhariwal J, et al. RSV-specific airway resident memory CD8+ T cells and differential disease severity after experimental human infection. Nat Commun 2015, 6, 10224. doi:10.1038/ncomms10224. 26687547 PMC4703893

[73] Meguro H , Kervina M, Wright PF. Antibody-dependent cell-mediated cytotoxicity against cells infected with respiratory syncytial virus: characterization of in vitro and in vivo properties. J Immunol 1979, 122, 2521–6. 571890

[74] Vangeti S , Periasamy S, Sun P, Balinsky CA, Mahajan AS, Kuzmina NA, et al. Serum Fc-mediated monocyte phagocytosis activity is stable for several months after SARS-CoV-2 asymptomatic and mildly symptomatic infection. Microbiol Spectr 2022, 10, e0183722. doi:10.1128/spectrum.01837-22. 36374040 PMC9769986

[75] Valkenburg SA , Zhang Y, Chan KY, Leung K, Wu JT, Poon LL. Preexisting antibody-dependent cellular cytotoxicity-activating antibody responses are stable longitudinally and cross-reactive responses are not boosted by recent influenza exposure. J Infect Dis 2016, 214, 1159–63. doi:10.1093/infdis/jiw346. 27493238 PMC5034955

[76] Blunck BN , Aideyan L, Ye X, Avadhanula V, Ferlic-Stark L, Zechiedrich L, et al. A prospective surveillance study on the kinetics of the humoral immune response to the respiratory syncytial virus fusion protein in adults in Houston, Texas. Vaccine 2021, 39, 1248–56. doi:10.1016/j.vaccine.2021.01.045. 33509697 PMC8152364

[77] Falsey AR , Singh HK, Walsh EE. Serum antibody decay in adults following natural respiratory syncytial virus infection. J Med Virol 2006, 78, 1493–7. doi:10.1002/jmv.20724. 16998887

[78] Xu S , Carpenter MC, Spreng RL, Neidich SD, Sarkar S, Tenney D, et al. Impact of adjuvants on the biophysical and functional characteristics of HIV vaccine-elicited antibodies in humans. npj Vaccines 2022, 7, 90. doi:10.1038/s41541-022-00514-9. 35927399 PMC9352797

[79] O’Donnell JS , Isaacs A, Jakob V, Lebas C, Barnes JB, Reading PC, et al. Characterization and comparison of novel adjuvants for a prefusion clamped MERS vaccine. Front Immunol 2022, 13, 976968. doi:10.3389/fimmu.2022.976968. 36119058 PMC9478912

