# Supplementary material for: Difference in respiratory syncytial virus-specific Fc-mediated antibody effector functions between children and adults
Source: Clin Exp Immunol. 2023 Aug 22;214(1):79–93. doi: 10.1093/cei/uxad101 (PMC10711356; doi:10.1093/cei/uxad101)
Supplement: uxad101_suppl_Supplementary_Figures [file uxad101_suppl_supplementary_figures.pdf]

Supplementary figure 1

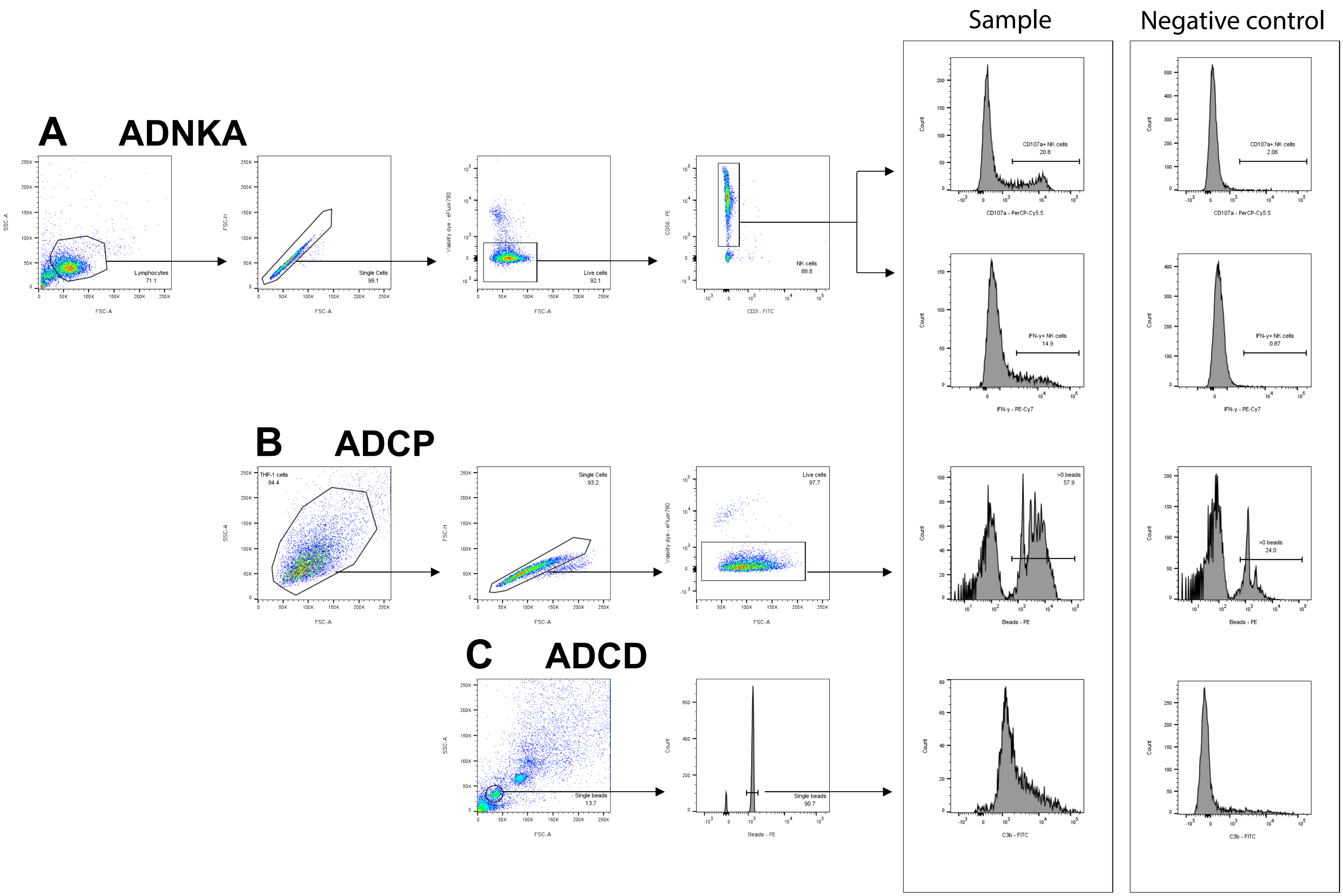

Supplementary figure S1. Gating strategy Fc-mediated antibody effector assays. (**A**) Gating strategy ADNKA. CD3-CD56+ NK cells were gated from the single, live cells gate. CD107a+ or IFN-γ+ NK-cells were subsequently gated. (**B**) Gating strategy ADCP. THP-1 cells were gated on forward and sideward scatter. Cells that have taken up one bead or more are selected from the single, live cells gate. (**C**) Gating strategy ADCD. Single beads were selected and geometric mean fluorescence of C3b-FITC was assessed. Dot plots and histograms are depicted for one representative sample. ADNKA, antibody-dependent NK cell activation; ADCP, antibody-dependent cellular phagocytosis; ADCD, antibody-dependent complement deposition; NK cells, natural killer cells.

Supplementary figure 2

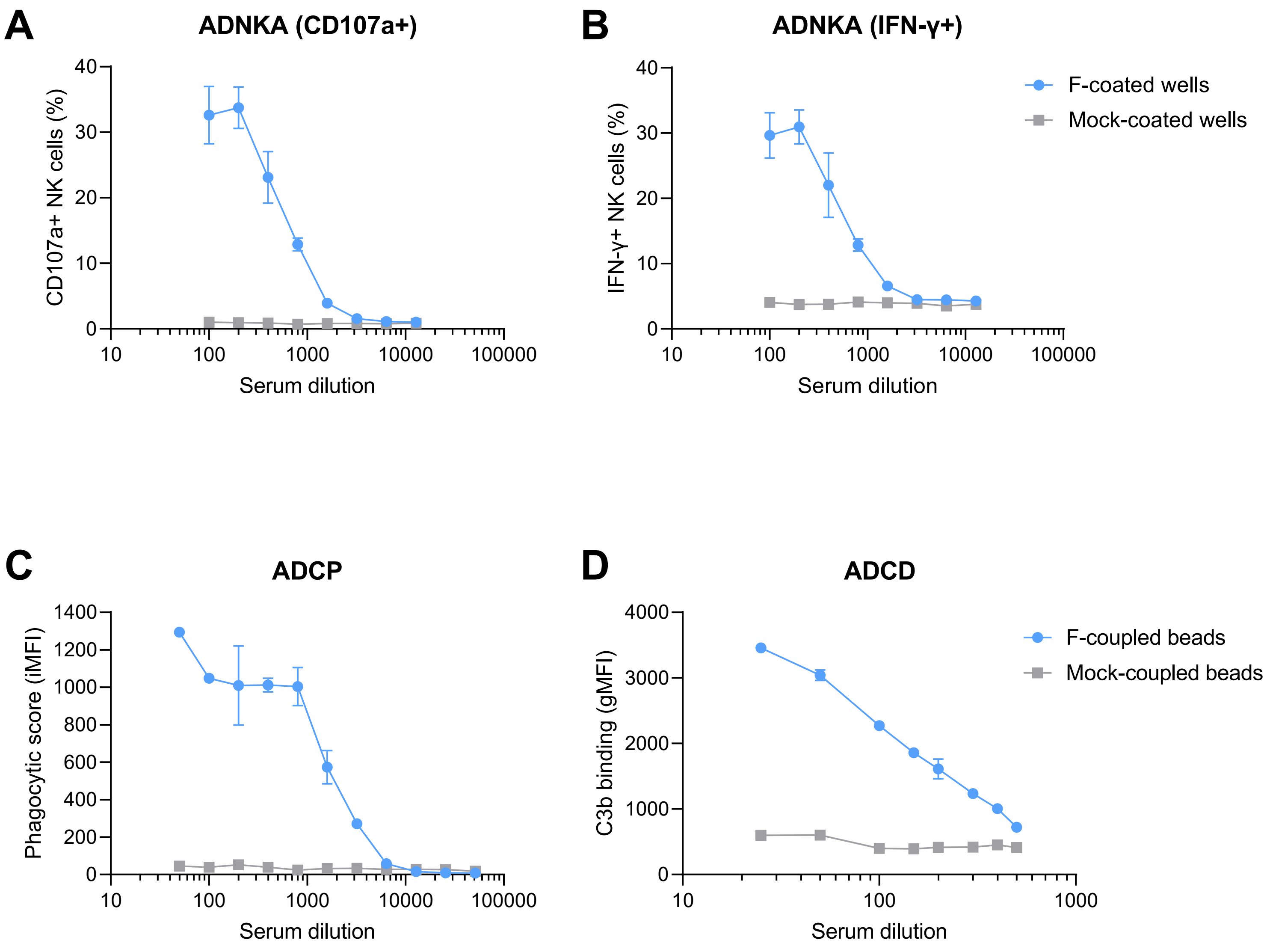

Supplementary figure S2. Serum titration curves for optimization of ADNKA, ADCP, and ADCD assays. Serum of 3 healthy adult donors was pooled and titrated to determine the optimal serum dilution for each assay. (**A**) ADNKA with percentage of CD107a+ NK cells as read-out. (**B**) ADNKA with percentage of IFN-γ+ NK cells as read-out. (**C**) ADCP by THP-1 cells with phagocytic score as read-out. (**D**) ADCD with gMFI as read-out. All data points represent means of technical duplicates with standard deviation. ADCD, antibody-dependent complement deposition; ADCP, antibody-dependent cellular phagocytosis; ADNKA, antibody-dependent NK cell activation; gMFI, geometric mean fluorescence intensity; iMFI, integrated mean fluorescence intensity; NK cells, natural killer cells.

Supplementary figure 3

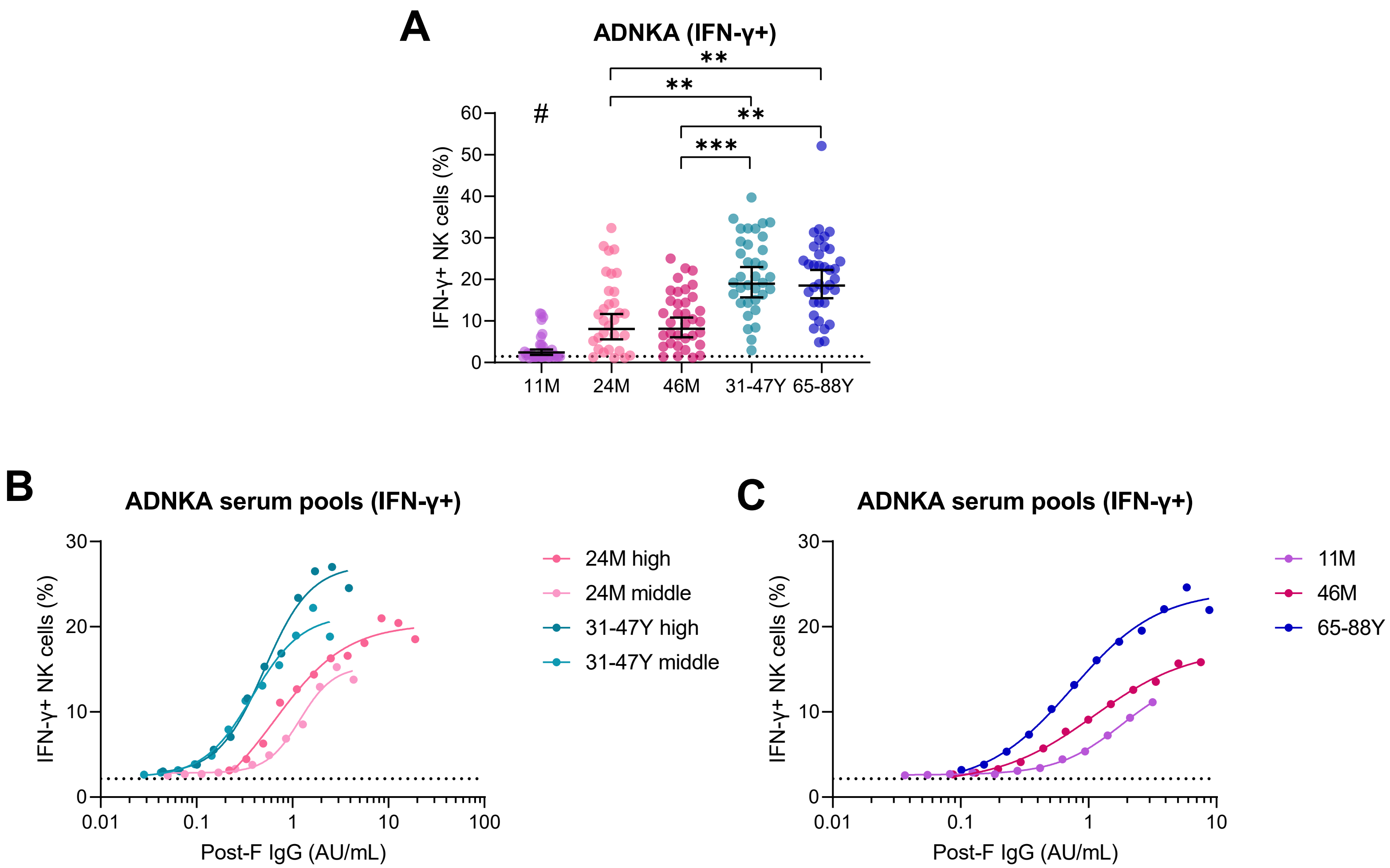

Supplementary figure S3. ADNKA with IFN-γ+ NK cells as read-out. (**A**) ADNKA with percentage of IFN-γ+ NK cells as read-out in different age groups: 11-months (n=33), 24-months (n=31), 46-months (n=35), adults (n=35), older adults (n=35)**.** All data points represent individual participants measured using NK cells from 3 healthy adult donors, and geometric means with 95% confidence intervals are depicted. Data is analyzed by Kruskal-Wallis test. # indicates statistical significance of at least $P<0.01$ compared to all other age groups. (**B-C**) Serum pools were prepared from the 10 individuals with the highest post-F-specific IgG concentration for each age group ("high") as well as pools of the 10 (adults) or 8 (24M) individuals with post-F-specific IgG levels around the GMC ("middle"). Post-F-specific IgG levels for each pool were measured with a multiplex immunoassay and ADNKA was assessed on a serial dilution range of each pool. (**B**) Graph shows ADNKA titration of 24-month-old "high" (pink), 24-month-old "middle" (light pink), adults "high" (teal), and adults "middle" (light teal) serum pools, with percentage of IFN-γ+ NK cells as read-out. (**C**) ADNKA titration of 11-month-old "high" (purple), 46-month-old "high" (dark pink), and older adults "high" (blue) serum pools, with percentage of IFN-γ+ NK cells as read-out. The average of three healthy NK cell donors is depicted. The curves are fitted based on a 4-parameter nonlinear regression model. The dotted lines indicate the level of the negative control. ADNKA, antibody-dependent NK cell activation; AU/mL, arbitrary units per milliliter; GMC, geometric mean concentration; NK cells, natural killer cells.

Supplementary figure 4

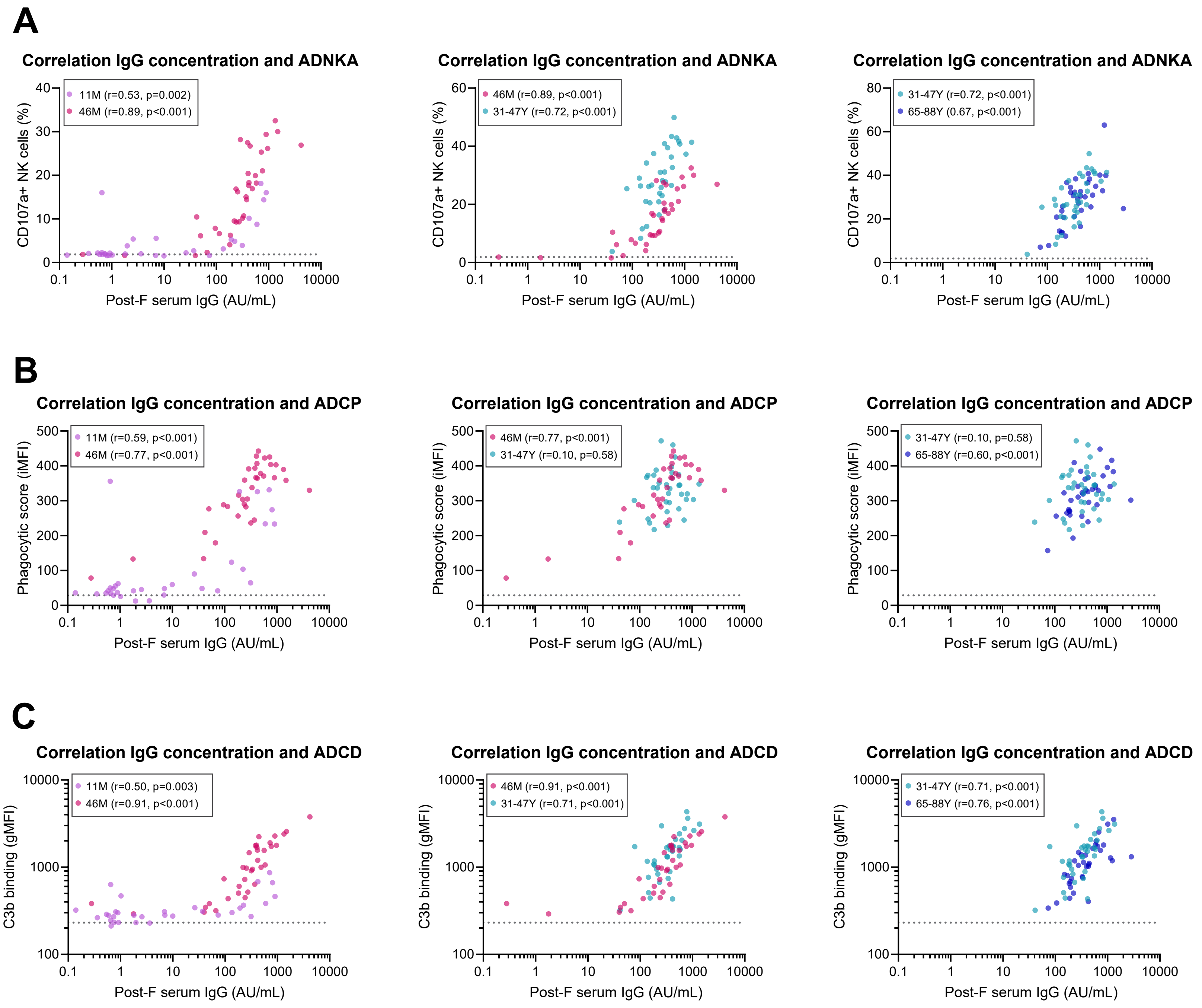

Supplementary figure S4. Correlation between post-F serum IgG and ADNKA, ADCP, and ADCD for different age groups. Post-F-specific serum IgG levels and ADNKA, ADCP, and ADCD were determined for 11-month-old (purple, n=33) and 46-month-old children (pink, n=35), adults (teal, n=35), and older adults (blue, n=35). Correlations are shown between post-F-specific serum IgG levels and (**A**) ADNKA measured as percentage CD107a+ NK cells, (**B**) ADCP by THP-1 cells with phagocytic score as read-out, and (**C**) ADCD with geometric mean fluorescence intensity as read-out. Dotted lines indicate negative control (ADNKA, ADCP, ADCD). All data points represent individual participants. Correlations are assessed using the Spearman method. ADCD, antibody-dependent complement deposition; ADCP, antibody-dependent cellular phagocytosis; ADNKA, antibody-dependent natural killer cell activation; AU/mL, arbitrary units per milliliter; gMFI, geometric mean fluorescence intensity; iMFI, integrated mean fluorescence intensity; NK cells, natural killer cells.

Supplementary figure 5

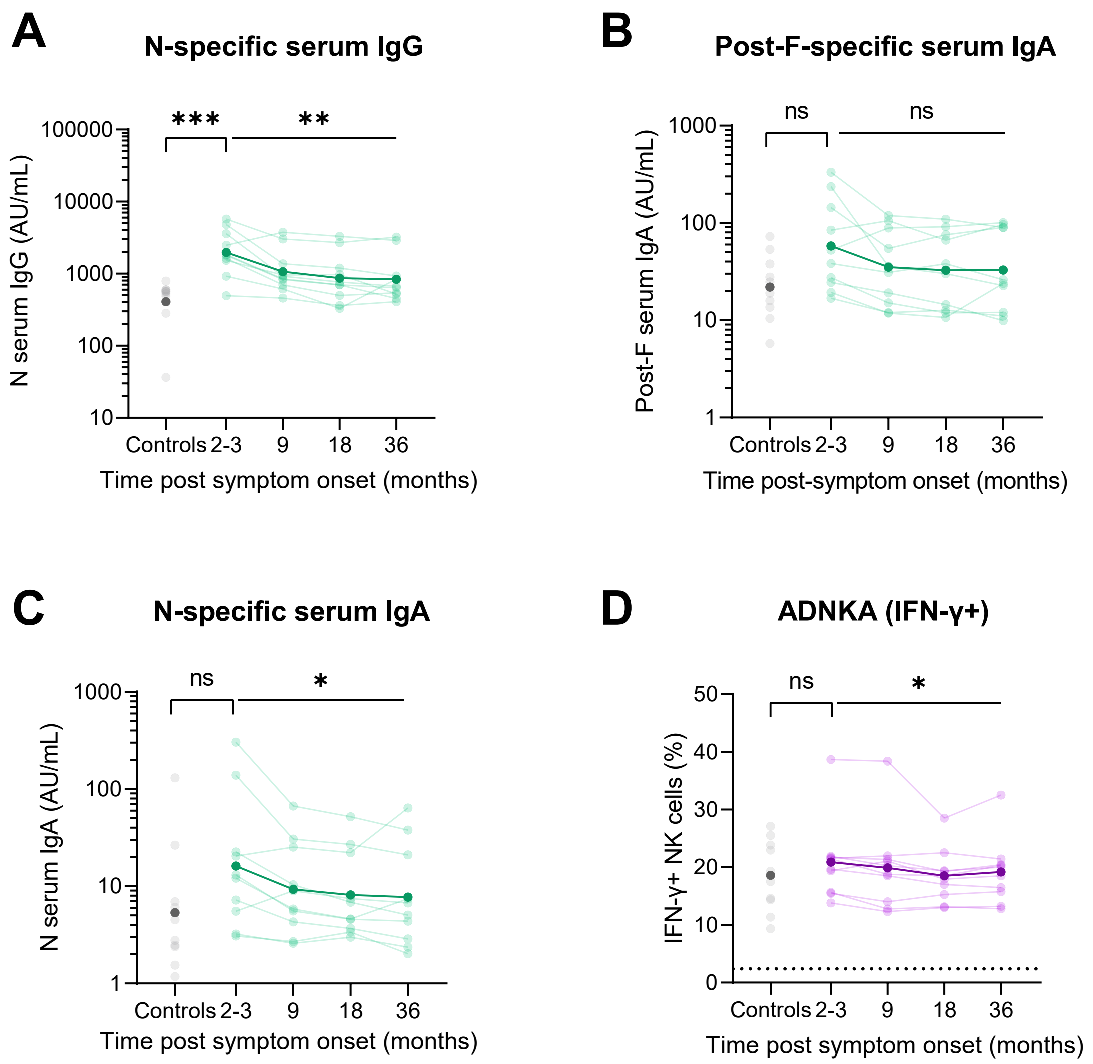

Supplementary figure S5. Kinetics post RSV infection of N- and post-F-specific serum IgG and IgA, and ADNKA with IFN-γ+ NK cells as read-out. (**A**) N-specific serum IgG concentrations, (**B**) post-F-specific serum IgA concentrations, and (**C**) N-specific serum IgA concentrations measured with a multiplex immunoassay in controls (n=10) and convalescent individuals followed over time after RSV infection (n=10). (**D**) ADNKA with percentage of IFN-γ+ NK cells as read-out in controls and convalescent individuals following RSV infection over time. Averages of three healthy NK cell donors are depicted. Dotted line indicates level of negative control. Each line represents a unique individual, dark lines indicate the geometric mean of all participants. Light grey dots represent individual age-matched controls, dark grey dots represent the geometric mean level of the control samples. Differences between controls and 2-3 months post symptom onset was assessed with a Mann-Whitney test. Differences over the complete follow-up time post infection were assessed with a Friedman test. *P<0.05; **P<0.01, ***P<0.001; ns, not significant. ADNKA, antibody-dependent NK cell activation; NK cells, natural killer cells.
